# Experimental and Numerical Investigation of Orthotropic CFR-PEEK and Ti-6Al-4V Osteosynthesis Plates Using a Multiscale Finite Element Modeling Approach

**DOI:** 10.3390/polym18212693

**Published:** 2026-10-30

**Authors:** Madina Isametova, Sholpan Kurmanova, Nurgul Seiitkazy, Yeszhan Ilyassov, Denis Tkachenko, Arailym Tursynbayeva

**Affiliations:** 1 Institute of Energy and Mechanical Engineering, Department of Mechanical Engineering, Satbayev University, Almaty 050000, Kazakhstan; isametova69@mail.ru (M.I.); yeszhan.ilyassov@gmail.com (Y.I.); d.tkachenko@satbayev.university (D.T.); 2 Institute of Architecture and Civil Engineering, Department of Civil Engineering and Building Materials, Satbayev University, Almaty 050000, Kazakhstan; sh.kurmanova@satbayev.university; 3 School of Engineering and Information Technology, META University, Almaty 050012, Kazakhstan; nurgulsejitkazy@gmail.com; 4 School of Transport Engineering and Logistics, Transport Engineering Direction, Satbayev University, Almaty 050000, Kazakhstan

**Keywords:** CFR-PEEK, carbon fibers, orthotropy, fused deposition modeling (FDM), Digimat-MF, finite element method (FEM), osteosynthesis plate, stress shielding, Tsai–Hill failure criterion

## Abstract

The application of carbon fiber-reinforced polyetheretherketone (CFR-PEEK) composites in osteosynthesis implants represents a promising approach to mitigating the stress-shielding effect associated with the high stiffness of conventional metallic implants. This study aimed to experimentally and numerically evaluate the effects of short carbon fiber content and the orthotropic mechanical properties of CFR-PEEK on the structural strength of osteosynthesis plates and load distribution within the bone–plate system. The investigation employed a multiscale approach integrating micromechanical modeling using the Mori–Tanaka homogenization method in Digimat-MF, experimental mechanical testing of FDM-fabricated CFR-PEEK specimens containing 10 wt.% carbon fibers, and finite element analysis of osteosynthesis plates made of CFR-PEEK with 10 and 30 wt.% carbon fibers and Ti-6Al-4V using MSC Patran/Marc. CFR-PEEK 30% specimens were neither fabricated nor experimentally tested. Their mechanical properties were predicted using Digimat-MF by applying correction factors derived from experimental data obtained for CFR-PEEK 10%. The results demonstrated that increasing the carbon fiber content from 10 to 30 wt.% enhanced the longitudinal elastic modulus. Experimental testing of CFR-PEEK containing 10 wt.% carbon fibers revealed an elastic modulus ranging from 1.76 to 3.10 GPa and a tensile strength of 47–54 MPa. Following process-related adjustments to the material properties, finite element simulations of the bone–plate system were performed under an axial load of 50 N. The calculated load shielding indices were 94.3% for Ti-6Al-4V, 28.6% for CFR-PEEK 10%, and 54.3% for CFR-PEEK 30%, indicating the potential of composite implants to improve mechanical load transfer to the surrounding bone tissue. A preliminary strength assessment based on the three-dimensional Tsai–Hill failure criterion yielded failure indices of 15.577 and 1.112 for CFR-PEEK 10% and CFR-PEEK 30%, respectively, under the assumed material strength parameters. Critical stress concentrations were predominantly localized around the fixation holes. These findings highlight the trade-off between reducing stress shielding and ensuring the structural integrity of composite osteosynthesis plates. The results further emphasize the need for localized optimization of fixation regions to improve mechanical reliability without increasing the overall implant thickness.

## 1. Introduction

Titanium alloys (Ti-6Al-4V) remain the gold standard for internal fracture fixation due to their excellent biocompatibility and mechanical strength [1,2,3,4,5]. However, their high elastic modulus (approximately 110 GPa) differs substantially from that of cortical bone, resulting in the stress-shielding effect, whereby the implant bears a disproportionate share of the mechanical load. This altered load distribution may contribute to bone resorption and atrophy over the long term [6,7,8,9].

Carbon fiber-reinforced polyetheretherketone (CFR-PEEK) has emerged as a promising alternative to metallic biomaterials for orthopedic fixation applications [10,11,12,13]. Unreinforced PEEK exhibits excellent radiolucency and biocompatibility; however, its relatively low elastic modulus (3–4 GPa) limits its applicability in highly loaded structural components. The incorporation of carbon fibers enables the mechanical strength and stiffness of the composite to be tailored [14,15], allowing its properties to be more closely matched to those of natural bone tissue [16,17,18]. This approach reduces the stiffness mismatch within the bone–plate system and promotes a more physiological distribution of mechanical loads, potentially supporting bone regeneration. CFR-PEEK has been successfully employed in the fabrication of osteosynthesis plates, screws, and intramedullary fixation devices [19,20,21].

A systematic review conducted by Chloros et al. [22], encompassing 361 patients with fractures involving the humerus, femur, radius, and ankle, reported high fracture union rates comparable to those achieved with conventional metallic fixation devices. Particular attention has been given to the clinical outcomes of distal radius fracture fixation. Randomized and prospective comparative studies evaluating CFR-PEEK and titanium volar locking plates, with follow-up periods of up to three years, revealed no statistically significant differences between the groups in wrist range of motion, Disabilities of the Arm, Shoulder and Hand (DASH) scores, Visual Analog Scale (VAS) pain scores, quality of fracture reduction, or fracture healing time [23,24].

In a multicenter study involving 71 clinical cases, the authors specifically highlighted the radiolucency of CFR-PEEK plates, which substantially facilitates intraoperative visualization of articular surfaces and subsequent radiographic monitoring of bone healing [25]. Collectively, the available clinical evidence supports the biomechanical suitability and radiographic advantages of CFR-PEEK as a viable alternative to titanium for internal fracture fixation.

Furthermore, investigations of distal radius fixation plates demonstrated that errors in computed tomography (CT)-based assessment of bone tissue parameters ranged from −3.2% to +2.6% for CFR-PEEK plates, compared with −30% to +67% for titanium plates [26]. These findings further highlight the potential advantages of CFR-PEEK not only in terms of mechanical performance but also for accurate postoperative imaging and monitoring of bone regeneration.

Despite the demonstrated clinical effectiveness of conventional CFR-PEEK osteosynthesis plates [27,28], their traditional mass production using injection molding or compression molding limits the ability to accommodate patient-specific anatomical characteristics. A promising solution to this limitation is the integration of additive manufacturing technologies, particularly fused deposition modeling (FDM), also referred to as fused filament fabrication (FFF). CT-based 3D printing enables the rapid fabrication of patient-specific implants designed to closely conform to the complex geometry and curvature of individual bone fragments. This approach can improve the congruence of bone–implant contact surfaces, reduce the risk of localized stress concentrations, and potentially shorten surgical procedures.

Furthermore, additive manufacturing using short carbon fiber-reinforced PEEK filament containing 30 wt.% carbon fibers (PEEK-30%CF) offers considerable opportunities for tailoring implant mechanical properties through design optimization. By adjusting the filament deposition paths and internal porosity, the local stiffness of the implant can be selectively controlled. This strategy enables the development of biomimetic structures with elastic moduli in the range of 18–24 GPa, approaching those of cortical bone. Such stiffness matching may substantially mitigate the stress-shielding effect, promote more physiological load transfer to the surrounding bone tissue, and potentially support natural bone regeneration.

Carbon fiber-reinforced PEEK composites for additive manufacturing are available in two main configurations: continuous carbon fiber-reinforced and short carbon fiber-reinforced materials. Although continuous fiber reinforcement provides superior longitudinal strength, short carbon fiber-reinforced filaments offer greater manufacturing flexibility for producing components with complex geometries, including screw holes and countersunk features. During extrusion, carbon fibers tend to align predominantly along the printing direction, while their possible partial reorientation may contribute to interlayer reinforcement. This phenomenon may potentially enhance interlaminar shear resistance and improve the mechanical strength of fixation regions in osteosynthesis plates [29].

To objectively assess the biomechanical performance of osteosynthesis implants manufactured from polymer-based composite materials, it is essential to consider the interrelationships among their microstructural characteristics, mechanical properties, and structural behavior under physiological loading conditions. In this context, multiscale modeling is particularly important, as it enables the prediction of effective composite properties from the characteristics of individual constituents and their subsequent integration into finite element analyses of the stress–strain response of the implant structure.

Finite element analysis (FEA) enables the evaluation of the stress–strain response of the bone–implant system, interfragmentary displacements, and the stress-shielding effect. Unlike Ti-6Al-4V, which is commonly modeled as an isotropic material, the mechanical properties of CFR-PEEK depend on the content, geometry, and orientation of the reinforcing carbon fibers. To determine the effective mechanical properties of the composite, the Digimat software (v2021) platform is employed, allowing the material microstructure to be incorporated into micromechanical models and its macroscopic mechanical behavior to be predicted for subsequent finite element analysis [30,31].

This approach is particularly relevant for additively manufactured carbon fiber-reinforced thermoplastics, as the mechanical properties of the final component are governed not only by the initial material composition but also by the processing parameters employed during fabrication. Digimat provides dedicated simulation tools for the additive manufacturing of polymers and composites, including fused filament fabrication/fused deposition modeling (FFF/FDM) processes, enabling the relationships among material characteristics, manufacturing conditions, and the mechanical behavior of printed components to be considered within an integrated computational framework [32,33,34].

It should be emphasized that the selection of an appropriate constitutive material model is a critical factor in ensuring the reliability and accuracy of finite element analysis (FEA) of CFR-PEEK structures. In several previously published FEA studies, carbon fiber-reinforced PEEK was modeled as a homogeneous, isotropic, linearly elastic material, with its mechanical behavior characterized by a single Young’s modulus and Poisson’s ratio, irrespective of the loading direction [35,36]. This approach considerably simplifies the numerical model and facilitates comparative assessments of implant materials; however, it does not fully account for the directional dependence of the mechanical properties of carbon fiber-reinforced composites.

Previous studies have demonstrated that CFR-PEEK exhibits orthotropic mechanical behavior due to the preferential orientation of reinforcing carbon fibers. However, some finite element studies still employ isotropic approximations to ensure comparability with previously published results [37]. In contrast to these approaches, the present study employs a three-dimensional orthotropic material model (3D Orthotropic) that accounts for the directional dependence of mechanical properties resulting from layer-by-layer material deposition during FDM printing and the preferential orientation of short carbon fibers. This approach enables a more comprehensive consideration of normal and shear stress components and allows the strength of the composite osteosynthesis plate to be evaluated using the Tsai–Hill failure criterion rather than the conventional von Mises criterion, which is primarily applied to isotropic materials.

Thus, in contrast to previous investigations based on isotropic representations of CFR-PEEK, the proposed approach explicitly accounts for the directional dependence of the mechanical properties of the carbon fiber-reinforced composite. This enables a more realistic assessment of the stress–strain distribution within the composite osteosynthesis plate, mechanical load transfer to the surrounding bone tissue, and interfragmentary micromotion when compared with a conventional isotropic Ti-6Al-4V fixation plate.

## 2. Materials and Methods

**Figure 1:**
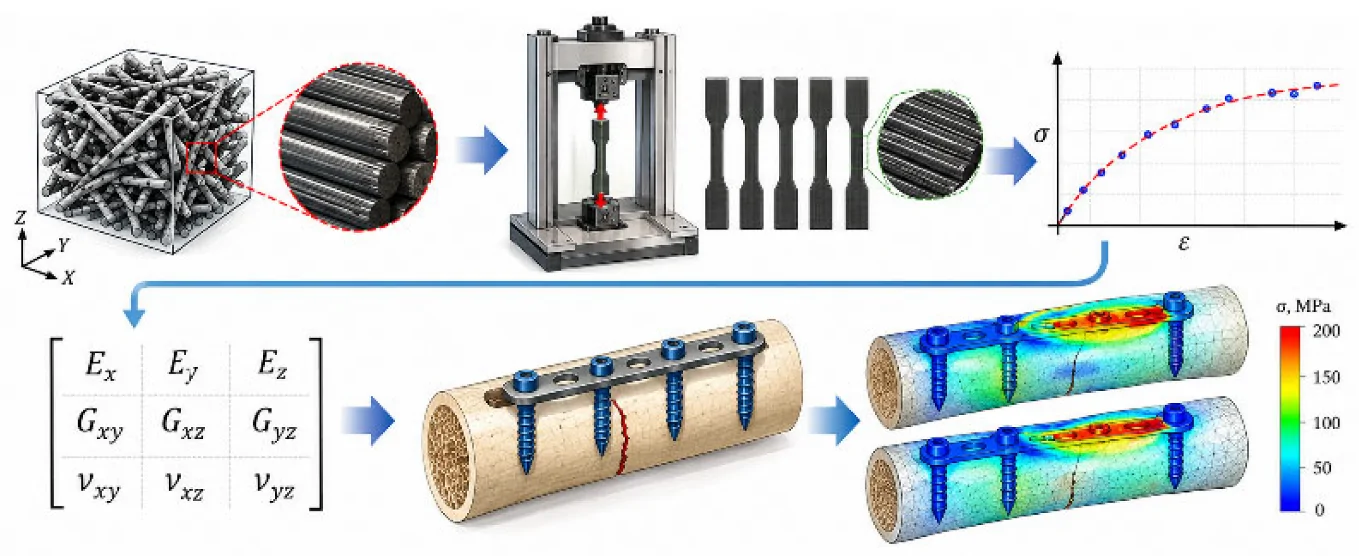
Schematic overview of the experimental–numerical investigation of the mechanical properties of carbon fiber-reinforced composites and the stress–strain response of the bone–plate system.

This study implements an integrated computational–experimental approach to the end-to-end design of composite materials based on multiscale modeling using the DIGIMAT software (v2021) platform. In the first stage, mechanical testing was performed on FDM-fabricated PEEK specimens reinforced with 10 wt.% short carbon fibers. A comparison between the experimentally measured and numerically predicted elastic moduli enabled the determination of correction factors accounting for the influence of the additive manufacturing process on the mechanical properties of the printed material. In the second stage, the derived correction factors were applied to the effective mechanical properties of PEEK (Dongguan Imai Intelligent Technology Co., Ltd., Dongguan, China) reinforced with 30 wt.% short carbon fibers (PEEK-30%CF), as predicted using DIGIMAT, for subsequent finite element analysis in MSC Patran (v2021). This methodology (Figure 1) establishes a sequential link between experimental characterization, micromechanical modeling of the composite microstructure, and macroscopic structural analysis of the osteosynthesis plate. The proposed approach enables the prediction of effective mechanical properties of carbon fiber-reinforced PEEK composites while accounting for manufacturing-induced effects. However, further experimental validation is required to establish the accuracy and reliability of the predicted material properties, particularly for composites with higher carbon fiber contents.

### 2.1. Overall Research Framework

The study comprised the following stages:

1. Micromechanical modeling of CFR-PEEK composites using the Digimat software platform.
2. Fabrication of CFR-PEEK specimens containing 10 wt.% carbon fibers using fused deposition modeling (FDM).
3. Experimental determination of the mechanical properties of the fabricated specimens.
4. Determination of correction factors and prediction of the effective orthotropic mechanical properties of CFR-PEEK containing 30 wt.% carbon fibers.
5. Development of a finite element model of the bone–fracture–plate–screw system.
6. Comparative analysis of the stress–strain response of osteosynthesis constructs made of CFR-PEEK 30% and Ti-6Al-4V.

This approach was adopted to account for the multiscale nature of the composite material under investigation, encompassing the properties of the polymer matrix and carbon fiber reinforcement at the constituent level and extending to the mechanical behavior of the final osteosynthesis construct at the structural level.

### 2.2. Mathematical Model

To comprehensively evaluate the mechanical behavior of CFR-PEEK osteosynthesis plates, a multiscale modeling approach was employed, integrating micromechanical modeling of the composite material, experimental calibration of its elastic properties, and finite element analysis of the bone–implant system. The mathematical framework is based on the Mori–Tanaka homogenization scheme, which enables the prediction of the effective mechanical properties of the composite by accounting for the content, geometry, and orientation of carbon fibers, together with the constitutive equations of orthotropic linear elasticity [38].

(1) $$C_{eff}=C_{m}+V_{f}\left(C_{f}-C_{m}\right):A_{f}$$

where

- $C_{eff}$—the effective stiffness tensor of the composite;
- $C_{m}$—the stiffness tensor of the PEEK matrix;
- $C_{f}$—the stiffness tensor of the carbon fibers;
- $V_{f}$—the volume fraction of carbon fibers;
- $A_{f}$—the strain concentration tensor of the reinforcing phase.

For a two-phase composite containing identically oriented ellipsoidal inclusions,

(2) $$A_{f}=T_{f}:{\left[\left(1-V_{f}\right)I+V_{f}T_{f}\right]}^{-1}$$

where

(3) $$T_{f}={\left[I+S:C_{m}^{-1}:(C_{f}-C_{m})\right]}^{-1}$$

Here, S denotes the Eshelby tensor, which accounts for the geometry of the inclusions, while I represents the fourth-order identity tensor.

For the comparative evaluation of the stress–strain response of osteosynthesis plates made of Ti-6Al-4V and CFR-PEEK, the von Mises equivalent stress was employed. This parameter enables the identification of stress concentration regions and facilitates the analysis of stress distribution patterns within the structures under identical loading conditions [39,40].

(4) $$\sigma_{vM}=\sqrt{\frac{1}{2}\left[{\left(\sigma_{x}-\sigma_{y}\right)}^{2}+{\left(\sigma_{y}-\sigma_{z}\right)}^{2}+{\left(\sigma_{z}-\sigma_{x}\right)}^{2}\right]+3(\tau_{xy}^{2}+\tau_{yz}^{2}+\tau_{zx}^{2})}$$

A three-dimensional stress state in a Cartesian coordinate system:

To assess the structural strength of CFR-PEEK osteosynthesis plates, the three-dimensional Tsai–Hill failure criterion was employed, accounting for the orthotropic mechanical properties of the composite material and the combined effects of normal and shear stresses. Unlike the von Mises criterion, the three-dimensional Tsai–Hill formulation accounts for differences in material strength along three mutually perpendicular directions, which is particularly important for carbon fiber-reinforced polymer composites manufactured using fused deposition modeling (FDM). The application of this criterion enables the assessment of potential failure initiation in the most highly stressed regions of the osteosynthesis plate, including fixation holes and areas of localized stress concentration.

For a three-dimensional stress state, one commonly used formulation of the Tsai–Hill failure criterion can be expressed as follows [41,42]:

(5) $${FI}_{TH}=\frac{\sigma_{1}^{2}}{X^{2}}+\frac{\sigma_{2}^{2}}{Y^{2}}+\frac{\sigma_{3}^{2}}{Z^{2}}-\left(\frac{1}{X^{2}}+\frac{1}{Y^{2}}-\frac{1}{Z^{2}}\right)\frac{\sigma_{1}\sigma_{2}}{2}-\left(\frac{1}{X^{2}}+\frac{1}{Z^{2}}-\frac{1}{Y^{2}}\right)\frac{\sigma_{1}\sigma_{3}}{2}-\left(\frac{1}{Y^{2}}+\frac{1}{Z^{2}}-\frac{1}{X^{2}}\right)\frac{\sigma_{2}\sigma_{3}}{2}+\frac{\tau_{12}^{2}}{S_{12}^{2}}+\frac{\tau_{23}^{2}}{S_{23}^{2}}+\frac{\tau_{13}^{2}}{S_{13}^{2}}$$

Here, $\sigma_{1}$, $\sigma_{2}$, $\sigma_{3}$ are the normal stress components along the local axes of the orthotropic material (not the principal stresses); $\tau_{12}$, $\tau_{23}$, $\tau_{13}$ are the shear stress components; X, Y, and Z denote the material strength limits in the corresponding directions; $S_{12}$, $S_{23}$, $S_{13}$ are the shear strength limits; and ${FI}_{TH}$ is the failure index calculated according to the Tsai–Hill criterion.

The failure condition is defined as follows:

(6) $$\{\begin{array}{c} {FI}_{TH}<1,\;\\ {FI}_{TH}\geq 1 \end{array}$$

In the first case, when ${FI}_{TH}<1$, the strength criterion is satisfied. In the second case, when ${FI}_{TH}\geq 1$, the material reaches or exceeds its predicted failure threshold.

The shear properties of CFR-PEEK were determined using a combined analytical and experimental approach [42] based on the transformation of the elastic constants of an orthotropic material. This method enables the estimation of the shear moduli ($G_{xy},G_{yz},G_{zx}$) from the elastic moduli obtained through uniaxial tensile testing of specimens oriented at 45° to the principal material axes in the corresponding planes. The calculations were performed using experimentally determined longitudinal elastic moduli, Poisson’s ratios, and tensile moduli measured under off-axis loading conditions.

(7) $$G_{xy}=\frac{1}{\frac{4}{E_{45,xy}}-\frac{1}{E_{x}}-\frac{1}{E_{y}}+\frac{2\nu_{xy}}{E_{x}}}\;G_{xz}=\frac{1}{\frac{4}{E_{45,xz}}-\frac{1}{E_{x}}-\frac{1}{E_{z}}+\frac{2\nu_{xz}}{E_{x}}}\;G_{yz}=\frac{1}{\frac{4}{E_{45,yz}}-\frac{1}{E_{y}}-\frac{1}{E_{z}}+\frac{2\nu_{yz}}{E_{y}}}$$

The application of this approach enables an indirect estimation of shear stiffness without the need for specialized shear testing. However, for FDM-printed composites, the effects of filament deposition orientation, interlayer adhesion, and process-induced anisotropy must be considered when assessing the applicability of the linear orthotropic elasticity model.

The integrated application of these models establishes a consistent relationship between the microstructure of CFR-PEEK, its macroscopic mechanical properties, and the biomechanical response of the implant, providing a framework for the comparative evaluation of CFR-PEEK osteosynthesis plates with different carbon fiber contents and conventional Ti-6Al-4V titanium alloy plates. The combined implementation of the Mori–Tanaka micromechanical homogenization model, experimental calibration of elastic properties, and orthotropic finite element modeling enables a systematic transition from the microstructural characteristics of CFR-PEEK to the mechanical behavior of the osteosynthesis construct. The von Mises equivalent stress is used for comparative analysis of stress distributions, whereas the Tsai–Hill failure criterion provides an assessment of the potential for failure initiation in the orthotropic composite material.

Thus, the proposed multiscale computational–experimental framework provides a methodological basis for investigating the influence of carbon fiber content on the mechanical properties of CFR-PEEK, stress distribution within the bone–implant system, interfragmentary micromotion, and the extent of stress shielding in comparison with conventional Ti-6Al-4V osteosynthesis plates.

### 2.3. Micromechanical Modeling of CFR-PEEK Using Digimat

In the Digimat software platform (Hexagon), the composite was modeled as a two-phase system consisting of a polyetheretherketone (PEEK) polymer matrix reinforced with short carbon fibers. The mechanical properties of the constituent materials, fiber content, fiber geometrical parameters, and spatial orientation, characterized by a fiber orientation tensor, were incorporated into the micromechanical model. Two composite formulations were investigated, PEEK-CF10 and PEEK-CF30, containing 10 wt.% and 30 wt.% carbon fibers, respectively. PEEK-CF10 was considered a moderately stiff composite with mechanical properties potentially approaching those of cortical bone, whereas PEEK-CF30 was evaluated as a highly reinforced composite exhibiting increased stiffness and potentially enhanced load-bearing capacity.

For both composite formulations, the constituent material properties (Table 1), fiber geometry, and orientation distribution were assumed to be identical. The carbon fiber weight fraction was the only parameter varied, allowing its specific influence on the predicted effective mechanical properties of the composite to be isolated.

**Figure 2:**
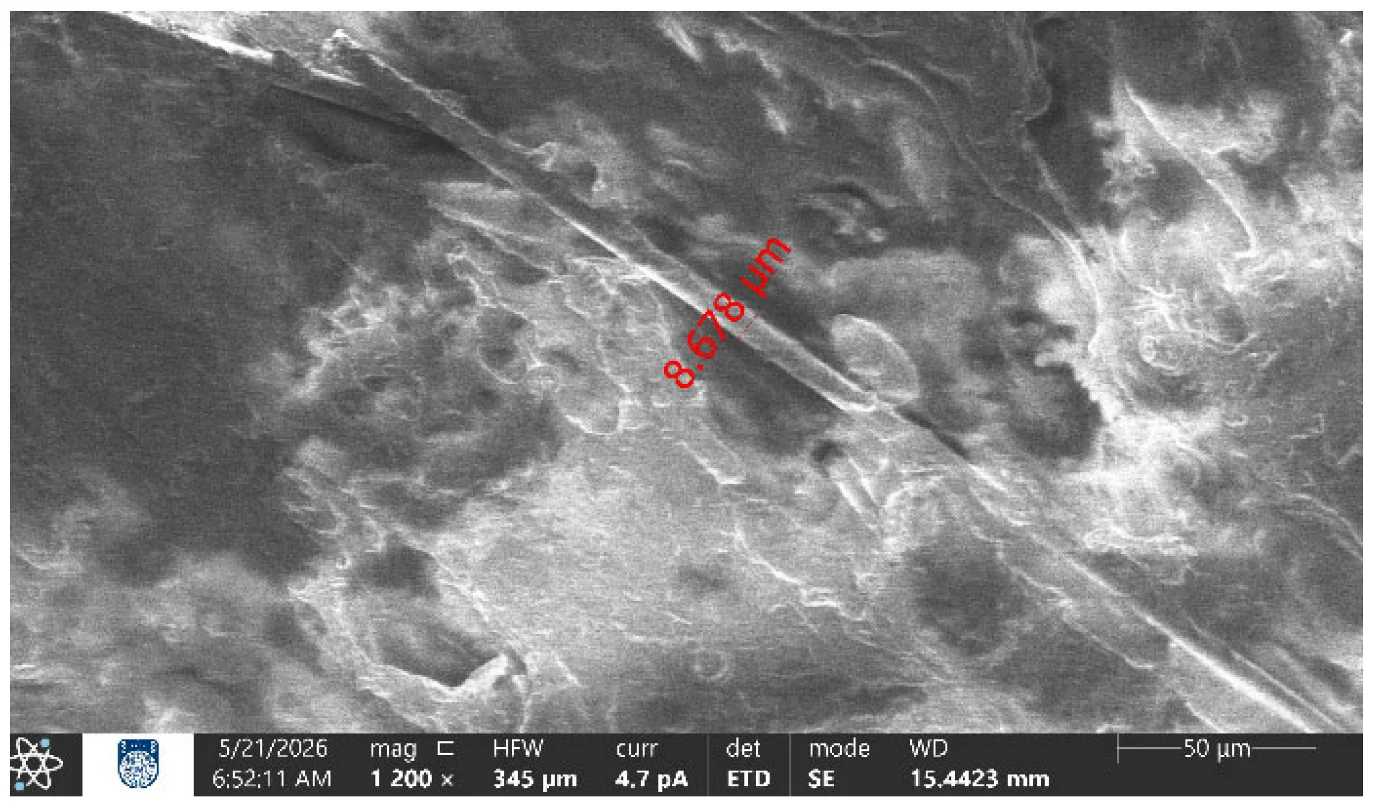
Scanning electron microscopy (SEM) image of carbon fibers within the PEEK-CF composite microstructure.

To develop the subsequent micromechanical model of the carbon fiber-reinforced composite in Digimat, it was necessary to determine the geometrical characteristics of the dispersed reinforcing phase. For this purpose, the microstructure of the composite material was examined using scanning electron microscopy (SEM Figure 2). The resulting SEM micrographs enabled visualization of the distribution of carbon fibers within the polymer matrix and determination of their characteristic geometrical dimensions.

Based on the specified constituent material properties and the microstructural data obtained from the SEM images, two micromechanical models were developed, differing in the content of the reinforcing phase. Short carbon fibers were used as the reinforcement and represented as elongated inclusions within the micromechanical model. The geometry of the reinforcing phase was characterized by the fiber aspect ratio, defined as the ratio of fiber length to diameter (L/D). An identical aspect ratio of L/D = 20 was assumed for both investigated composite formulations, PEEK-CF10 and PEEK-CF30. Keeping this parameter constant eliminated the influence of variations in fiber geometry, allowing the specific effect of carbon fiber weight fraction on the effective mechanical properties of the composite to be investigated.

The spatial orientation of the carbon fibers was described using a second-order fiber orientation tensor. In the numerical model, the fibers were assumed to be preferentially aligned along material direction 1 (X-axis), corresponding to the primary filament deposition direction during fused filament fabrication (FFF). For an idealized unidirectional fiber orientation distribution, the orientation tensor was defined as follows:

(8) $$T=\left[\begin{array}{ccc} a_{11} & a_{12} & a_{13}\\ a_{21} & a_{22} & a_{23}\\ a_{31} & a_{32} & a_{33} \end{array}\right]$$

The modeling parameters for the two CFR-PEEK composite configurations are presented in Table 2.

**Figure 3:**
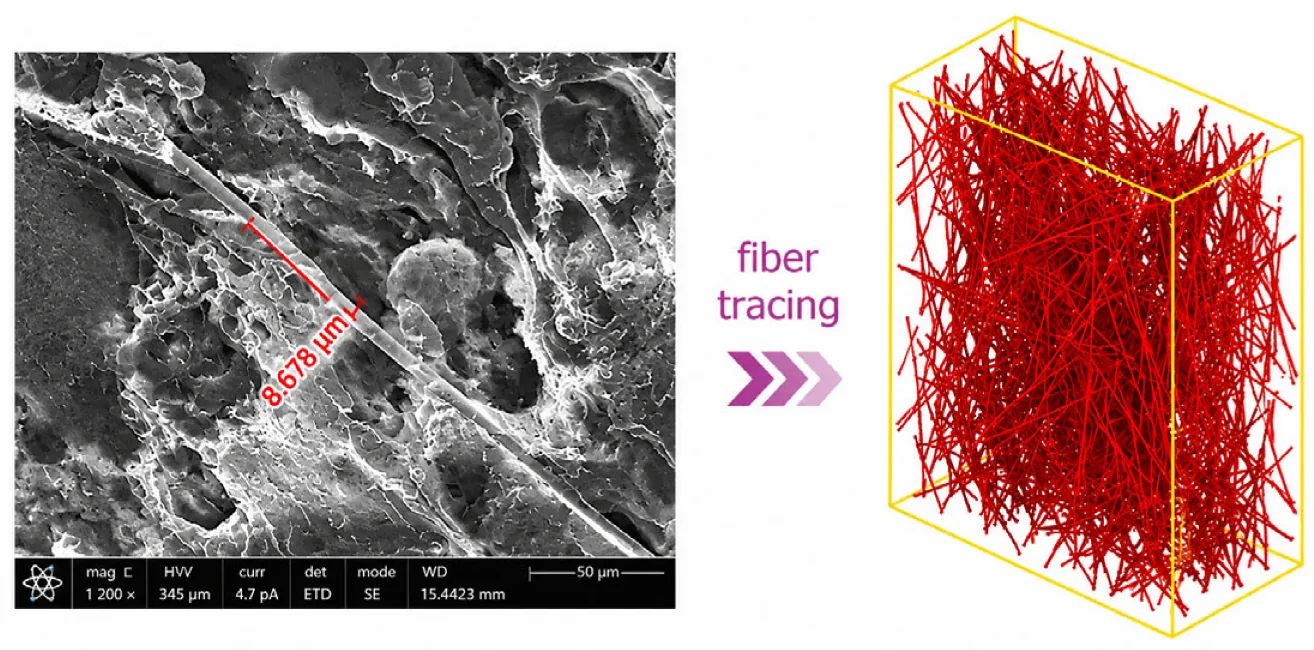
Microstructure of carbon fiber-reinforced PEEK and schematic three-dimensional visualization of the short carbon fiber distribution.

Figure 3 presents a reconstruction of the microstructure of the carbon fiber-reinforced PEEK (CFR-PEEK) composite based on SEM image analysis and numerical modeling of the spatial distribution of short carbon fibers.

### 2.4. Experimental Determination of the Mechanical Properties of PEEK-CF10 and Validation of the Micromechanical Model

To comparatively validate the results of micromechanical modeling performed in Digimat, mechanical tests were conducted on CFR-PEEK 10% specimens fabricated using fused filament fabrication/fused deposition modeling (FFF/FDM). The experimental investigations aimed to assess the agreement between the effective mechanical properties predicted by Digimat and the actual properties of the carbon fiber-reinforced composite after additive manufacturing.

Additionally, the influence of printing parameters, including material deposition orientation, on the strength and stiffness of the fabricated specimens was investigated. This enabled the assessment of changes in the mechanical properties of the composite induced by the additive manufacturing process and provided a basis for incorporating process-induced anisotropy when defining the orthotropic properties of CFR-PEEK in subsequent finite element modeling of the osteosynthesis plate.

For the experimental investigations, tensile test specimens (were fabricated according to the dimensions specified in Table 3, in compliance with GOST 11262–2017 (ISO 527-2:2012) [43]. Three-dimensional models of the specimens were prepared for subsequent FFF/FDM fabrication.

**Figure 4:**
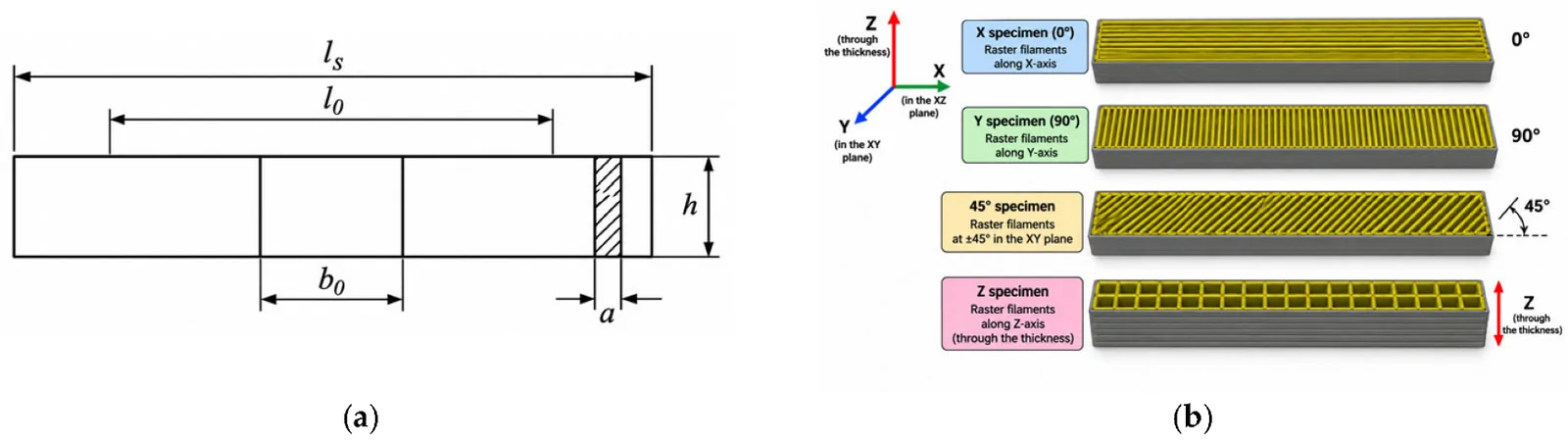
Geometry and printing orientations of tensile test specimens: (**a**) schematic representation of a Type 1 specimen in accordance with GOST 11262–2017 (ISO 527-2:2012), showing the characteristic dimensions of the test specimen; (**b**) schematic illustration of FDM-fabricated specimens with different printing orientations (0°, 90°, 45°, and Z-direction), demonstrating the orientation of the specimens relative to the build coordinate system.

To experimentally determine the direction-dependent mechanical properties of the composite, specimens were manufactured with printing orientations of 0°, 90°, and along the Z-axis (build direction in Figure 4). Mechanical testing was performed to determine the directional elastic moduli and tensile strengths required to characterize material anisotropy and provide input data for subsequent finite element analysis.

To investigate the influence of FDM printing parameters on the mechanical strength of carbon fiber-reinforced composite components, a full factorial experimental design was developed involving three independent variables: nozzle temperature, printing speed, and layer thickness. Experimental investigations were conducted on specimens fabricated using different combinations of these processing parameters to identify their effects on the mechanical strength of the printed material and determine optimal printing conditions. The experimental design matrix is presented in Table 3.

The experimentally determined elastic moduli were used to evaluate the degree of material anisotropy and subsequently compared with the effective elastic properties predicted by micromechanical modeling in DIGIMAT.

**Figure 5:**
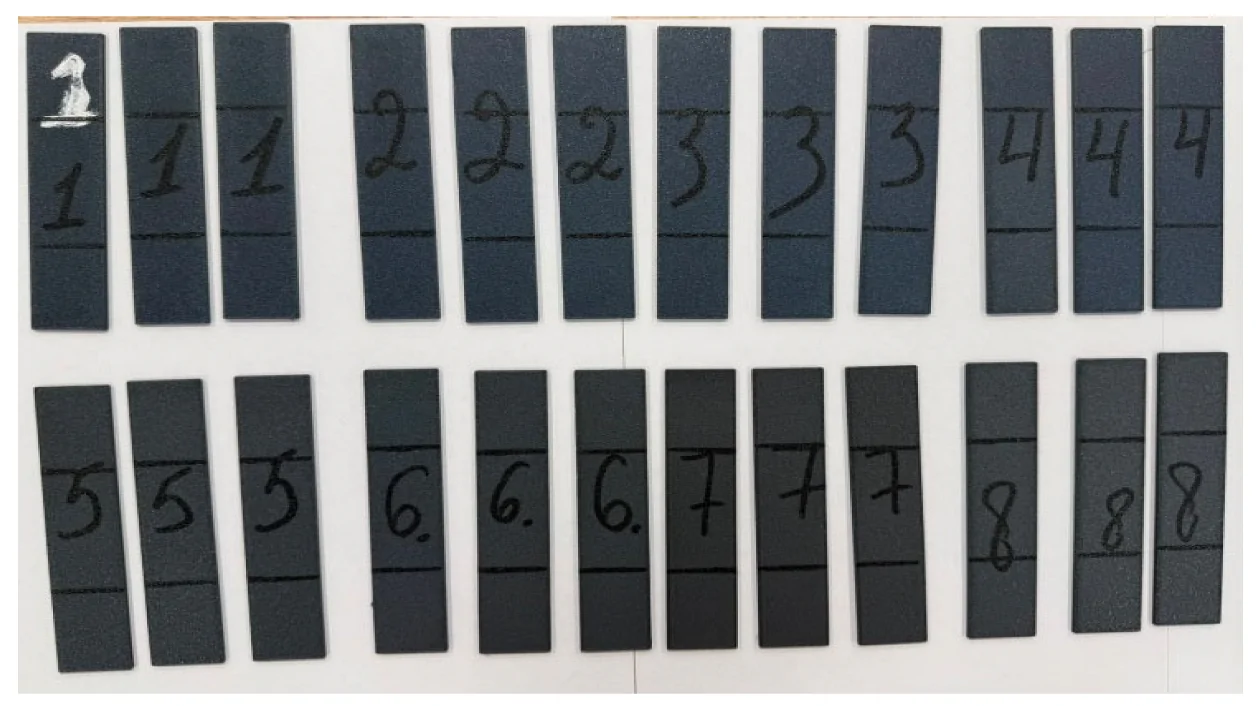
FDM-fabricated carbon fiber-reinforced polymer specimens comprising eight experimental sets, with three specimens per set.

A total of eight experimental sets, each comprising three carbon fiber-reinforced polymer specimens, were fabricated using different combinations of the printing parameters specified in Table 3, resulting in 24 specimens. Photographs of the fabricated specimens are presented in Figure 5. All specimens were manufactured using a Bambu Lab X1E 3D printer equipped with an Automatic Material System (AMS).

### 2.5. Tensile Testing of PEEK-CF10 Specimens

**Figure 6:**
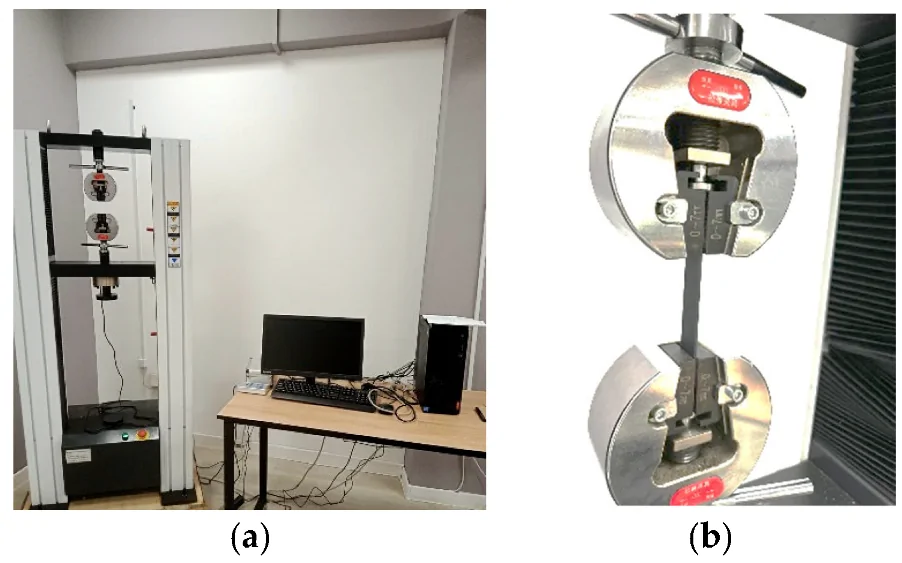
Experimental setup for tensile testing using the WDW-50E microcomputer-controlled electronic universal testing machine: (**a**) general view of the testing machine and data acquisition system; (**b**) close-up view of a polymer composite specimen mounted in the tensile grips prior to testing.

Tensile tests were conducted at Satbayev University (Kazakh National Research Technical University named after K. I. Satpayev) using a WDW-50E microcomputer-controlled electronic universal testing machine (Figure 6). Prior to testing, the cross-sectional dimensions of each specimen were measured individually and entered into the testing machine’s control software to ensure accurate calculation of engineering stress. The tensile tests were performed at a constant crosshead displacement rate of 20 mm/s.

The tensile stress–strain curve was obtained by applying a progressively increasing tensile load to the specimen until failure. The crosshead displacement rate was set to 2.0 ± 5.0 mm/min. The elongation at break was calculated from the measured extension at failure (Δl) relative to the initial gauge length of the specimen.

### 2.6. Finite Element Modeling of the Bone–Plate System Using Ti-6Al-4V and CFR-PEEK

To comparatively evaluate the biomechanical performance of osteosynthesis constructs, finite element (FE) simulations of the bone–plate system were performed using fixation plates made of Ti-6Al-4V and CFR-PEEK. Under identical model geometries, boundary conditions, and external loading conditions, the influence of the plate material on the stress–strain response of the bone tissue and implant, load distribution, and interfragmentary micromotion was investigated.

Ti-6Al-4V was modeled as an isotropic material, whereas CFR-PEEK was characterized by orthotropic mechanical properties derived from micromechanical modeling performed in Digimat. To account for the potential degradation of composite mechanical properties during the FFF/FDM manufacturing process, an additional correction factor was introduced. This factor was determined by comparing the mechanical properties of CFR-PLA predicted using Digimat with those obtained from experimental testing of additively manufactured specimens. The correction factor was subsequently applied to the predicted mechanical properties of CFR-PEEK to approximate the effects of additive manufacturing and evaluate how process-induced reductions in material properties influence the biomechanical response of the bone–plate system.

Bone fixation plates are among the most widely used implants for internal fracture fixation [44]. Their primary advantages include providing mechanical stability to fractured bone segments and the ability to withstand various types of mechanical loading, including tension, compression, shear, bending, and torsion. These implants are commonly employed in open reduction and internal fixation (ORIF) procedures, as well as in bridge plating techniques.

**Figure 7:**
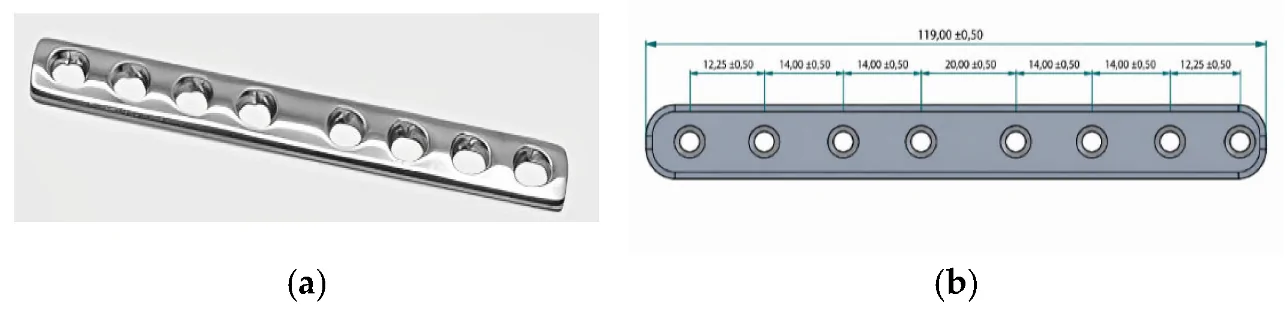
Dynamic compression plate (DCP) for fixation of radial and ulnar fractures: (**a**) general view of the osteosynthesis plate; (**b**) schematic representation of the plate geometry and fixation hole arrangement used for subsequent finite element modeling.

Modern osteosynthesis plates are available in a wide range of geometrical configurations, including anatomically contoured and periarticular designs, and incorporate holes for locking and non-locking screws. The selection of an appropriate plate configuration (Figure 7) depends on the anatomical location, fracture morphology, and specific biomechanical requirements of the fixation procedure [45].

A standard 3.5 mm dynamic compression plate (DCP) was used as the reference fixation construct. The plate had a straight configuration with oval holes designed to accommodate 3.5 mm cortical screws.

To simplify the numerical analysis, the cross-section of the radial diaphysis was approximated as a hollow cylinder with an outer diameter of 15.9 mm and an inner diameter of 6.37 mm. These dimensions fall within the anatomical range of the adult human radial shaft.

A three-dimensional geometric model of the bone–plate–screw system was developed using Autodesk Inventor and subsequently imported into MSC Patran for finite element analysis. To simulate the mechanical interactions between the structural components, 16 contact pairs were defined, with a contact search tolerance of 0.001 m. The prescribed contact conditions enabled load transfer between the components of the fixation system and facilitated the investigation of stress and strain distributions within the bone tissue and implant.

The model was discretized using four-node tetrahedral solid elements, which are suitable for approximating complex three-dimensional geometries [46,47]. The characteristic element size was set to 0.002 m. To assess the numerical accuracy of the solution, a mesh independence study was performed. The results demonstrated that further mesh refinement resulted in variations of less than 5% in the maximum von Mises equivalent stress, indicating acceptable mesh convergence under the selected simulation conditions.

**Figure 8:**
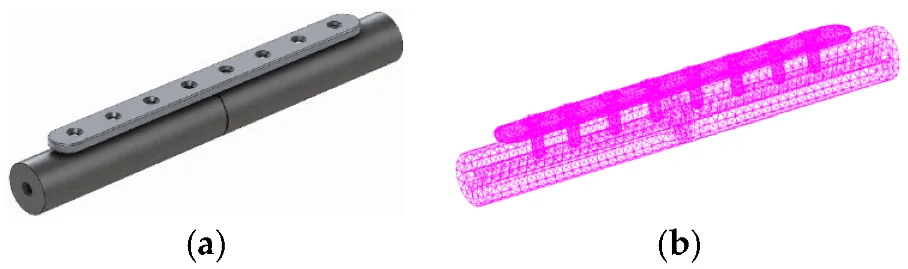
Finite element model of the bone–plate assembly: (**a**) three-dimensional geometric model of the bone–plate assembly; (**b**) finite element mesh of the bone–plate assembly.

The finite element model of the bone–plate–screw system is presented in Figure 8.

The generated finite element mesh consisted of 43,624 elements. To improve numerical accuracy in regions of stress concentration, local mesh refinement was applied around the fracture site, plate fixation holes, and contact interfaces between the screws, bone, and plate. The resulting mesh provided an adequate representation of the structural geometry and enabled a numerically stable solution for evaluating the stress–strain response of the bone–implant system.

Axial compression was selected as the primary loading condition for the comparative biomechanical assessment of the bone–implant system. This loading configuration was chosen to investigate mechanical load transfer between the bone tissue and fixation plate, evaluate the stress-shielding effect, and assess mechanical stability at the fracture site. Axial compression provides a suitable basis for identifying the influence of stiffness differences between the Ti-6Al-4V titanium alloy and CFR-PEEK composite on stress distribution, deformation, and interfragmentary micromotion under identical loading conditions.

**Figure 9:**
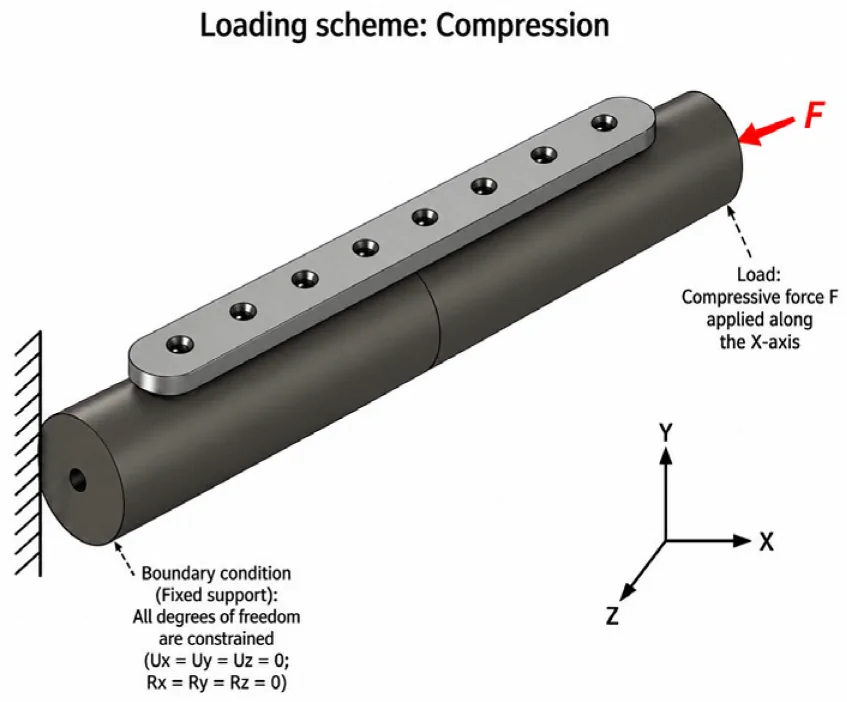
Axial compression loading scheme and boundary conditions of the bone–plate assembly.

To simulate the forces acting on the radius and the osteosynthesis implant during daily activities in patients who have undergone reconstructive surgery, a load of 50 N was applied. The applied force of 50 N provides a reasonable approximation of the physiological mechanical loading conditions experienced by the upper limb. Unlike the bones of the lower extremities, the radius is not a primary weight-bearing structure, and its loading conditions during the early stages of postoperative recovery are predominantly determined by passive forearm muscle tone, finger movements, and light daily hand activities. A load of 50 N is commonly used as a baseline loading condition in orthopedic biomechanics to evaluate the fixation of distal radius fractures, as supported by previous studies [48]. A load of 50 N was applied to the distal end of the radius, while the proximal end was fully constrained in all degrees of freedom, preventing both translational and rotational displacements. The prescribed loading and boundary conditions ensured the comparability of the numerical results obtained for the two implant materials and enabled the evaluation of load redistribution within the bone–plate–screw system. The loading configuration and corresponding boundary conditions are illustrated in Figure 9.

The fracture geometry was modeled according to the AO/OTA classification system. A simple transverse diaphyseal fracture of the radius, classified as type 22-A3 [49], was selected for the investigation. This fracture type is characterized by a fracture line oriented approximately perpendicular to the longitudinal axis of the bone, with an inclination angle of less than 30°.

To reproduce the fracture configuration, the bone was divided into two fragments separated by a 1 mm interfragmentary gap. The adopted geometry enabled the evaluation of stress distributions within the bone tissue and implant, as well as the relative displacement of bone fragments under axial loading. The use of identical fracture geometry ensured the comparability of finite element simulation results obtained for osteosynthesis constructs incorporating CFR-PEEK and Ti-6Al-4V fixation plates.

### 2.7. Materials

In the finite element simulations, cortical bone was modeled as a homogeneous, isotropic, linearly elastic material, with mechanical properties representative of healthy cortical bone in a young adult. This assumption is widely adopted in finite element studies of orthopedic implants and provides a consistent basis for comparing different fixation configurations under identical modeling conditions.

In reality, cortical bone exhibits anisotropic mechanical behavior, with its properties depending on the orientation of the underlying microstructure. Consequently, the use of an isotropic material model may influence the absolute values of local stresses and strains. Nevertheless, because identical bone material properties were assigned to all investigated configurations, this simplification was considered acceptable for the comparative assessment of the mechanical behavior of the fixation plates, although its influence on the quantitative results cannot be entirely excluded [50].

The mechanical properties of all materials used in the finite element simulations are summarized in Table 4.

This approach enables the evaluation of the stress–strain response of the bone–plate system using physiologically representative mechanical properties of cortical bone. For subsequent finite element analysis of the osteosynthesis plate, a CFR-PEEK composite containing 30 wt.% carbon fiber reinforcement was selected. The effective orthotropic properties of the material were initially determined through micromechanical modeling using DIGIMAT software. Since the computational model primarily represents the properties of the consolidated composite without fully accounting for manufacturing defects introduced during FFF printing, the predicted properties will be adjusted to account for the effects of the additive manufacturing process.

## 3. Results

### 3.1. Results of DIGIMAT Modeling of the Mechanical Properties of CFR-PEEK Composites with 10% and 30% Carbon Fiber Content

To characterize the mechanical behavior of the composites, the principal parameters of the orthotropic material model, including Young’s moduli $E_{1},E_{2},E_{3},G_{12},G_{13},G_{23}$, were determined.

The effective elastic properties of CFR-PEEK composites containing 10 and 30 wt.% carbon fibers were evaluated using Digimat-MF software (v2021), which implements micromechanical homogenization methods. The initial properties of the constituent materials were defined based on the published literature and microstructural analysis.

The main parameters used to develop the micromechanical models in Digimat-MF are presented in Table 5.

**Figure 10:**
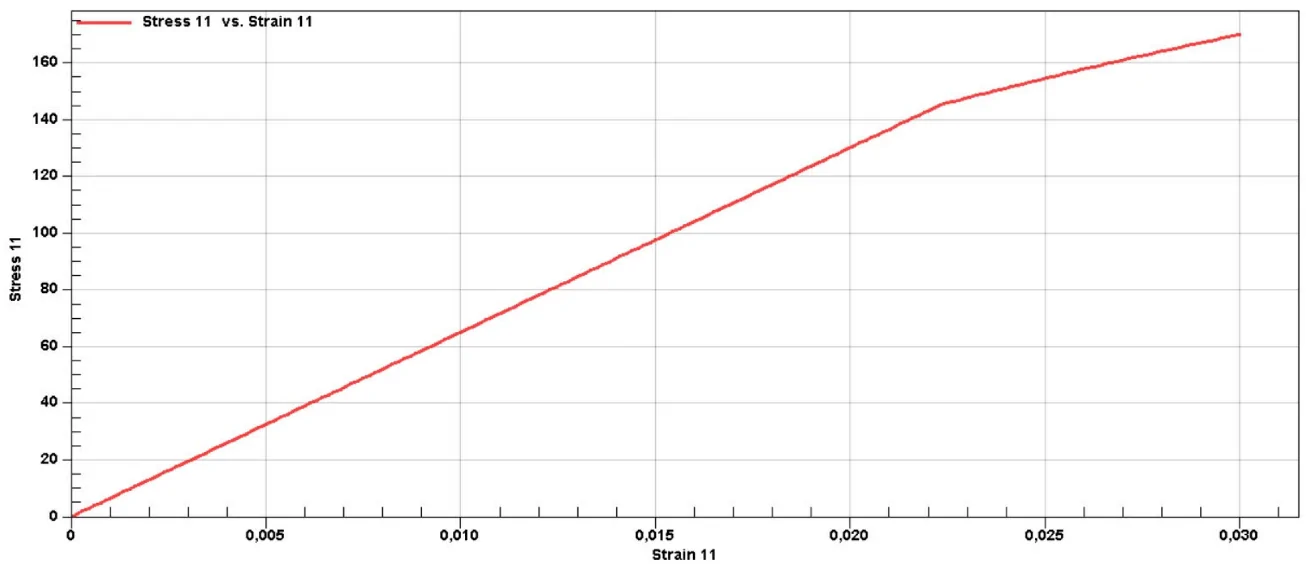
Stress–strain curve of the CFR-PEEK composite containing 10 wt.% carbon fibers.

Figure 10 presents the corresponding stress–strain curve, illustrating the mechanical response of the investigated CFR-PEEK composite containing 10 wt.% carbon fibers. The obtained results serve as a baseline for subsequent evaluation of the influence of the printing process on the effective mechanical properties of the material and for defining material parameters in finite element simulations.

Figure 10 presents the stress–strain curve of the CFR-PEEK composite containing 10 wt.% carbon fibers. Within the strain range of 0–0.020, the stress increases almost linearly from 0 to approximately 280 MPa, indicating predominantly elastic behavior. At a strain of approximately 0.025, the stress reaches 340 MPa, while at the maximum plotted strain of 0.030 (3%), it approaches 400 MPa. Based on the slope of the initial linear region, Young’s modulus is estimated to be approximately 14.0–14.1 GPa, which is consistent with the calculated value of E_1_ = 14.06 GPa. A slight deviation from linearity at strains exceeding approximately 2% indicates a change in material stiffness under further loading.

The Digimat simulations provide tabulated effective engineering properties of the composite, including Young’s moduli, shear moduli, and Poisson’s ratios. These results (Table 6) characterize the elastic behavior of the material along different directions and are subsequently used to define the orthotropic material properties in finite element simulations.

**Figure 11:**
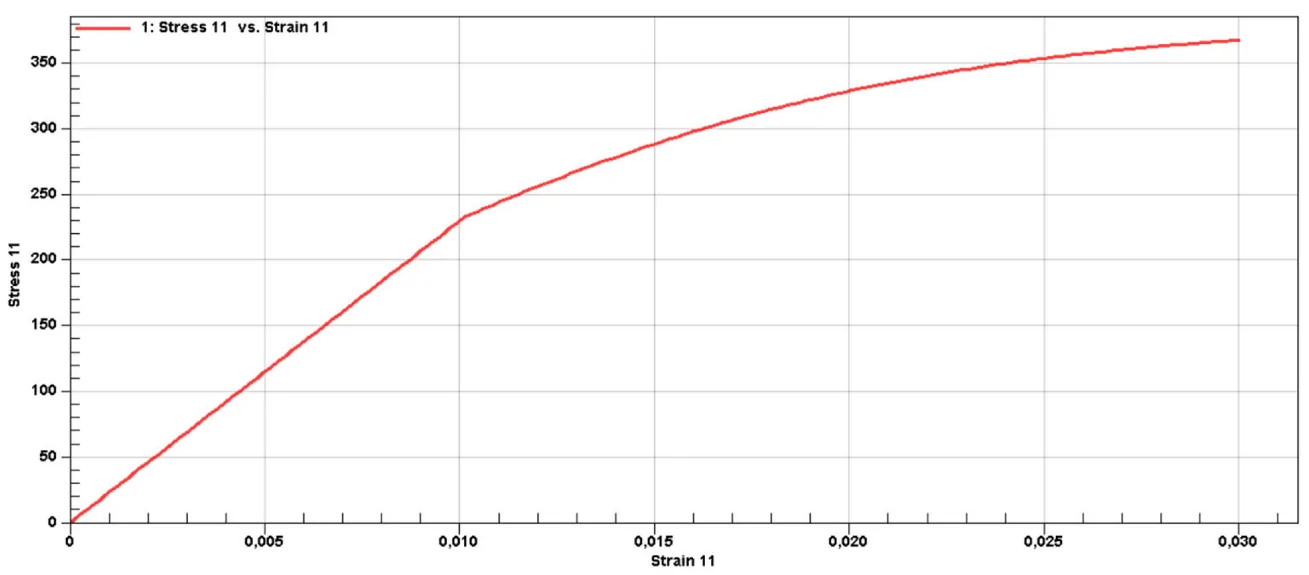
Stress–strain curve of the CFR-PEEK composite containing 30 wt.% carbon fibers.

Similarly, the effective engineering properties of the CFR-PEEK composite containing 30 wt.% carbon fibers were calculated using Digimat software. The predicted Young’s moduli, shear moduli, and Poisson’s ratios were subsequently used for comparative analysis and finite element modeling. The predicted stress–strain curve and the corresponding effective engineering properties of the composite containing 30 wt.% carbon fibers are presented in Figure 11 and Table 7, respectively.

The stress–strain curve demonstrates an approximately linear relationship within the investigated strain range. At a strain of approximately 1%, the stress reaches 350 MPa, increasing to about 730 MPa at 2% strain and approximately 1050 MPa at the maximum strain of 3%. Based on the slope of the linear region, Young’s modulus is estimated to be approximately 35–36 GPa, indicating a substantial increase in composite stiffness as the carbon fiber content increases from 10 to 30 wt.%.

### 3.2. Experimental Tensile Testing Results of CFR-PEEK with 10 wt.% Carbon Fibers

#### Tensile Strength Along the Printed Layers

**Figure 12:**
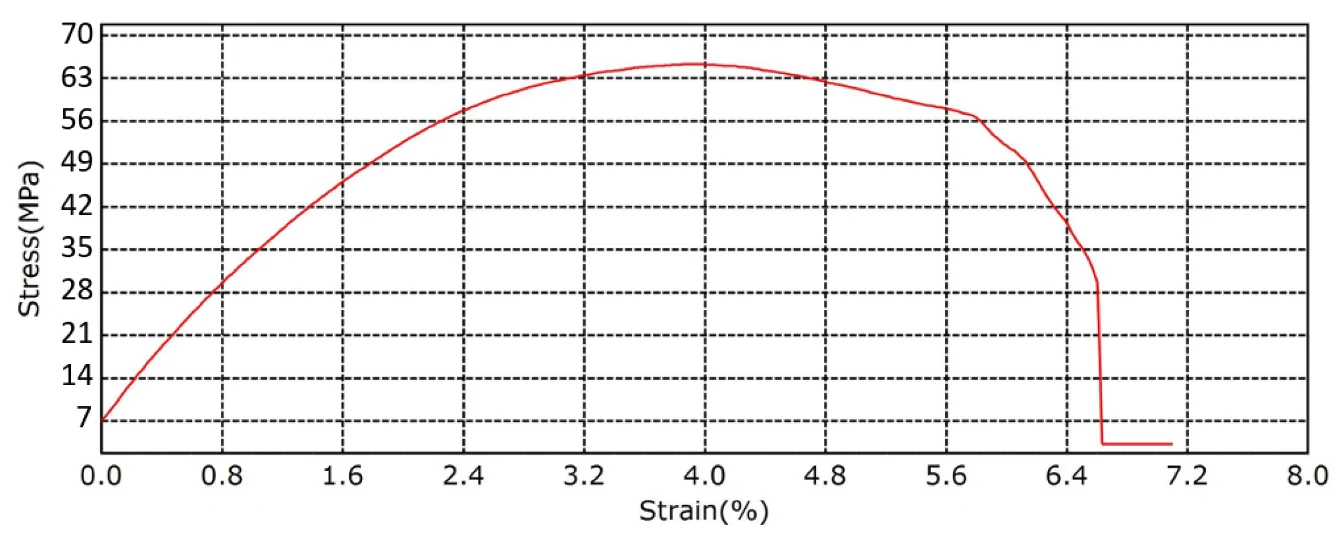
Stress–strain curve of CFR-PEEK containing 10 wt.% carbon fibers under tensile loading to failure for the fourth specimen.

The stress–strain curves obtained during tensile testing to failure are presented in Figure 12. Photographs of the specimens before and after fracture are shown in Figure 9.

The specimen dimensions were as follows: width—20 mm, thickness—2 mm, overall length—120 mm, and gauge length—50 mm. The testing speed was 2.00 mm/s. The material tested was carbon fiber-reinforced PEEK (CFR-PEEK).

Figure 12 shows the carbon fiber-reinforced polymer composite specimens after uniaxial tensile testing.

**Figure 13:**
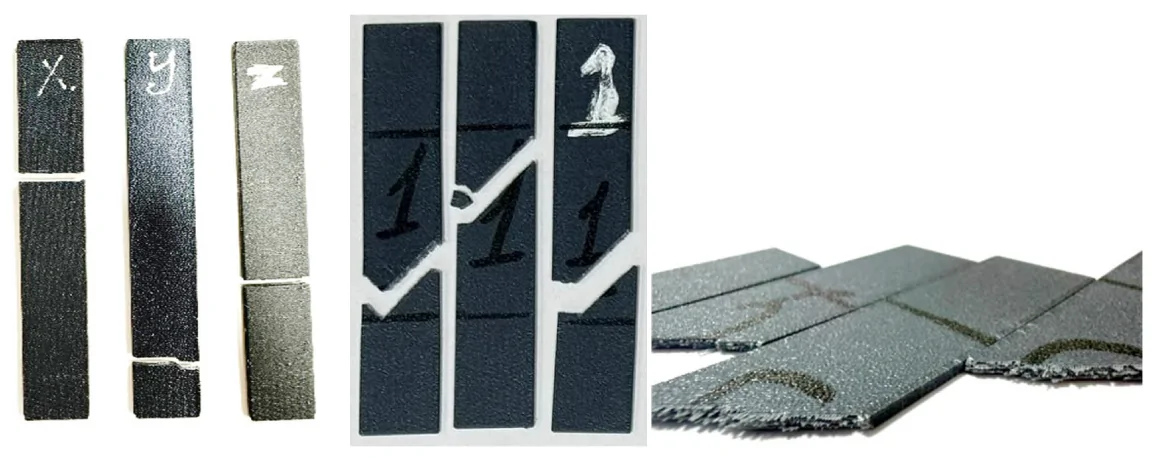
Specimens after tensile fracture.

Specimens fabricated with printing orientations along the X, Y, and Z axes exhibited predominantly transverse fracture, with fracture lines oriented approximately perpendicular to the longitudinal axis of the specimens (Figure 13). In contrast, specimens printed with a 45° raster orientation demonstrated inclined fracture patterns characterized by diagonal and stepped fracture lines. The magnified image reveals irregularities on the fracture surfaces and localized material separation along inclined regions. These differences in fracture behavior may be attributed to the orientation of the printing paths, interlayer bonding characteristics, and the combined effects of normal and shear stresses under tensile loading.

To evaluate the mechanical properties of the 3D-printed carbon fiber-reinforced composite, uniaxial tensile tests were performed. The main mechanical properties, including Young’s modulus (E), maximum tensile stress (σ_m_), and elongation at break (δ), were determined from the obtained stress–strain curves. Testing multiple specimens enabled the assessment of variability in the measured properties and the influence of additive manufacturing characteristics on material strength and stiffness. The results of the mechanical tests are presented in Table 8.

A statistical analysis of the experimental results was performed, and the regression coefficients were determined. The first-order regression model incorporating interaction effects is expressed as follows:

(9) $$Y=b_{0}+b_{1}X_{1}+b_{2}X_{2}+b_{3}X_{3}+b_{12}X_{1}X_{2}+b_{13}X_{1}X_{3}+b_{23}X_{2}X_{3}$$

To accurately calculate the regression coefficients, the variables must be transformed into coded variables:

(10) $$x_{1}=\frac{T-390}{10}$$

(11) $$x_{2}=\frac{h-0.22}{0.06}$$

(12) $$x_{3}=\frac{V-30}{10}$$

where

T=380–400 °C
h=0.16–0.28 mm
V=20–40 mm/s

Regression Equation for Tensile Strength at Break

After calculating the effects, the following equation is obtained:

(13) $$Y_{\sigma }=50+1.25x_{1}-0.25x_{2}+1.25x_{3}-1.5x_{1}x_{2}-0.5x_{1}x_{3}+1.25x_{1}x_{3}x_{2}$$

Coefficient of Determination (R^2^):

(14) $$R^{2}\approx 0.8$$

Statistical analysis of the experimental data using a full $2^{3}$ factorial design yielded first-order regression models incorporating two-factor interactions. The coefficient of determination for tensile strength at break was $R^{2}\approx 0.8$, indicating a high goodness of fit and demonstrating the potential of the developed model to predict the mechanical properties of the investigated material within the studied factor ranges.

**Figure 14:**
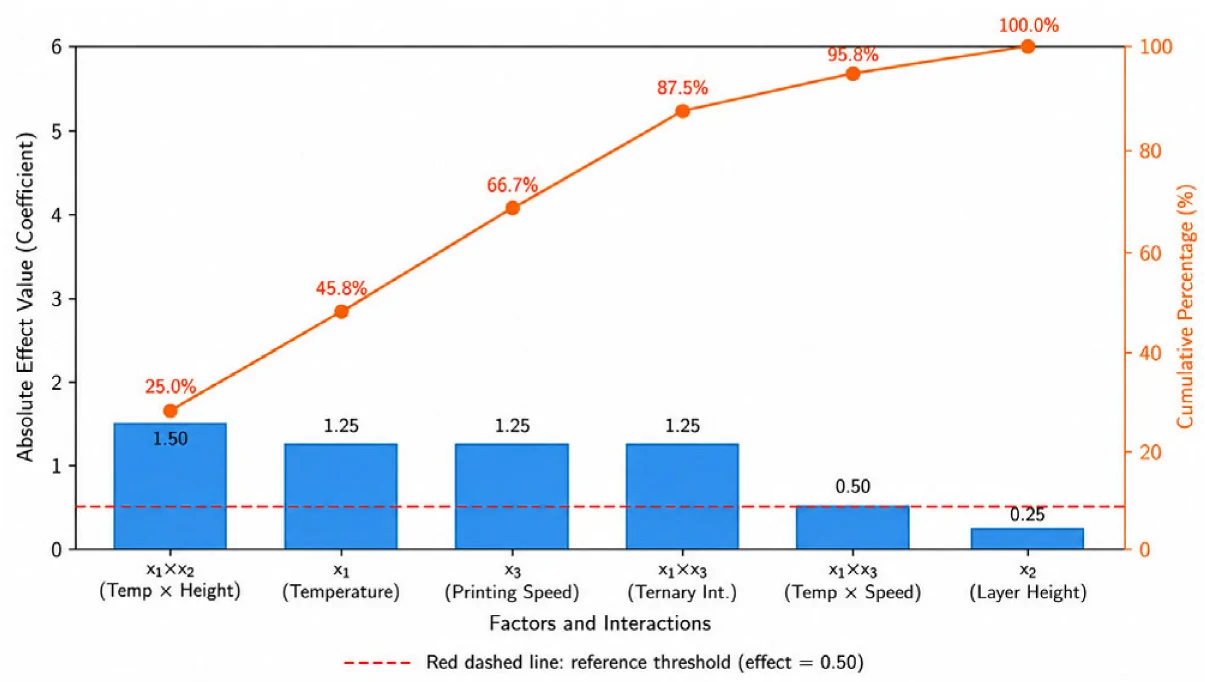
Pareto Chart of Effects on Tensile Strength.

To identify the most significant factors and their interactions, a Pareto chart (Figure 14) was constructed to rank the effects of extrusion temperature, printing speed, and layer thickness according to their influence on material strength. The obtained results were used to identify the most influential processing parameters and select appropriate printing conditions.

The presented Pareto chart illustrates the ranking of FDM printing process parameters according to their influence on the tensile strength of CFR-PEEK specimens fabricated using FDM technology. The interaction between extrusion temperature and layer thickness makes the largest contribution, accounting for 25.0%. This is followed by extrusion temperature, printing speed, and the three-factor interaction among extrusion temperature, layer thickness, and printing speed, each accounting for 20.8%. The interaction between extrusion temperature and printing speed accounts for approximately 8.3%, whereas layer thickness exhibits a relatively minor individual effect (4.2%). The interaction between layer thickness and printing speed was excluded from the final model due to its statistical insignificance, as determined using Lenth’s method. These findings highlight the crucial role of the combined and interactive effects of processing parameters in determining the strength characteristics of CFR-PEEK composites.

**Figure 15:**
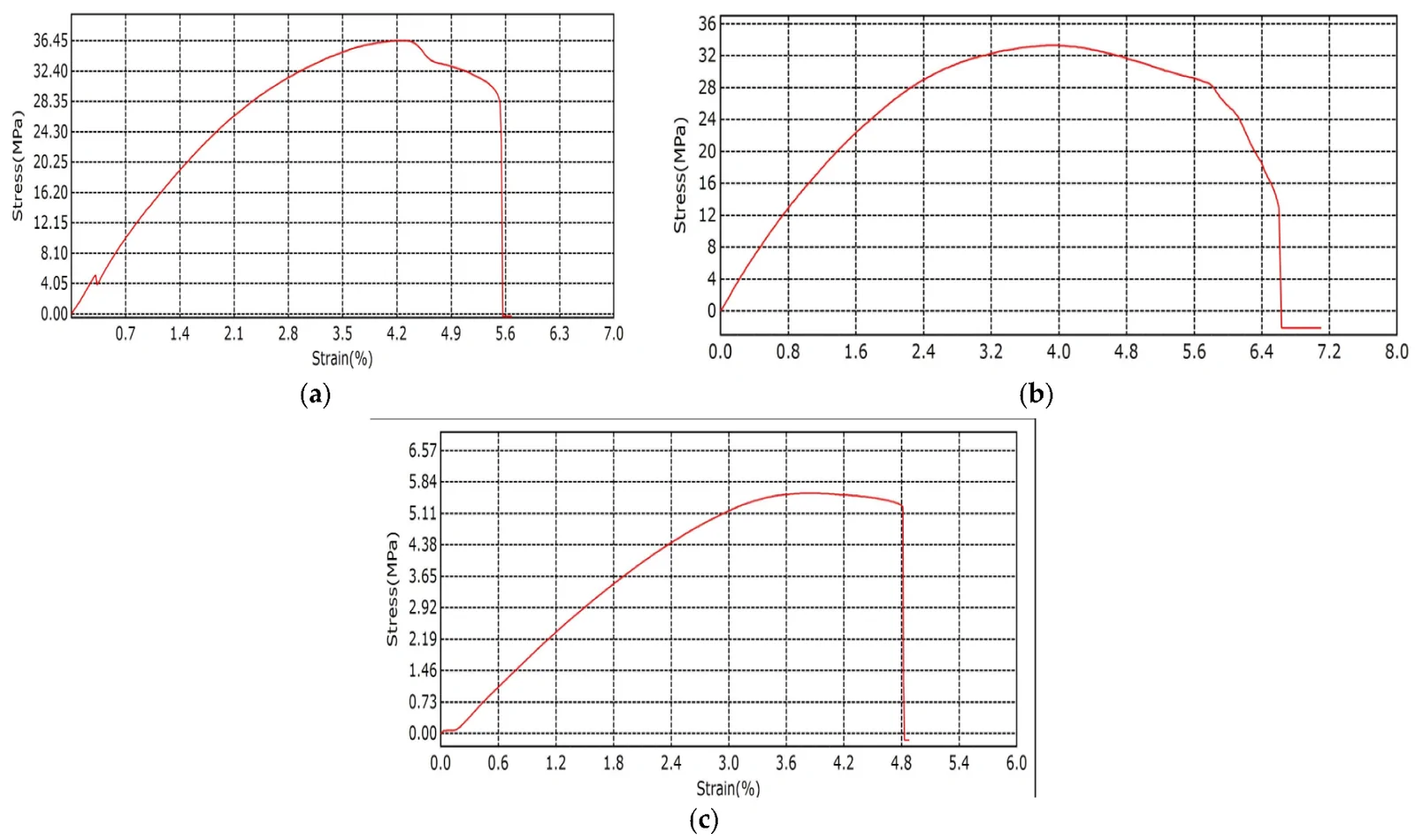
Stress–strain curves of CFR-PEEK containing 10 wt.% carbon fibers under tensile loading to failure: (**a**) along the Y-axis and (**b**) along the z-axis; (**c**) 45° represents two different specimen configurations.

The stress–strain curves (Figure 15) used to determine the mechanical properties of the carbon fiber-reinforced composite in the transverse direction (Y-axis, Figure 15a) and through-thickness direction (Z-axis, Figure 15b) are presented below. The corresponding mechanical properties are summarized in the following table.

Both curves exhibit nonlinear stress–strain behavior, characterized by an initial increase in stress, followed by a gradual reduction in load-bearing capacity and subsequent failure. For specimens tested along the Y-axis (Figure 15a), the maximum tensile stress reaches approximately 34 MPa at a strain of 3.8–4.0%, with failure occurring at approximately 5.1% strain. In contrast, specimens tested along the Z-axis (Figure 15b) exhibit a slightly lower maximum tensile stress of approximately 33 MPa, while the strain at failure increases to approximately 6.6%. These results indicate comparable tensile strengths in both specimen configurations, although the Z-axis specimens demonstrate greater deformation capacity before failure. The shear moduli of the CFR-PEEK composite were estimated using the analytical relationships presented in Equation (7), based on the elastic moduli obtained from tensile tests and the corresponding Poisson’s ratios. This approach enabled an indirect evaluation of the shear stiffness under the assumption of linear orthotropic elastic behavior. The observed differences suggest that the printing orientation influences the deformation and failure behavior of FDM-printed CFR-PEEK composites, potentially reflecting variations in interlayer bonding and internal structural organization.

The mechanical properties of the carbon fiber-reinforced composite determined experimentally in the transverse direction (Y-axis) and through-thickness direction (Z-axis) are summarized in Table 9 below.

The experimental results obtained are consistent with findings reported in previous studies investigating the mechanical properties of carbon fiber-reinforced composites fabricated using FDM technology. The measured Young’s modulus values and the observed trends in mechanical behavior are in agreement with those reported in the literature [51], supporting the comparability of the present experimental findings with previously published results.

### 3.3. Adjustment of Composite Mechanical Properties to Account for Additive Manufacturing Process Parameters

The mechanical properties of CFR-PEEK were predicted using DIGIMAT software based on the Mori–Tanaka micromechanical model, incorporating the properties of the PEEK polymer matrix as well as the content, geometry, and orientation of carbon fibers.

To account for the effects of the FDM printing process on the elastic properties of the composite, a correction factor was determined by comparing the predicted and experimentally measured Young’s moduli of CFR-PEEK containing 10 wt.% carbon fibers. The resulting average correction factor of 0.595 was rounded to 0.60 and subsequently applied to predict the mechanical properties of CFR-PEEK containing 30 wt.% carbon fibers.

This approach assumes that the relative influence of the printing process remains comparable when the polymer matrix and reinforcing fiber types are unchanged. However, transferring the correction factor between different fiber contents represents a modeling assumption that requires further experimental validation.

The mechanical properties of carbon fiber-reinforced composites predicted using micromechanical homogenization may differ from experimentally measured values due to the characteristics of additive manufacturing, including fiber orientation, interlayer adhesion, porosity, and printing parameters. Previous studies comparing mean-field homogenization predictions with experimental results have reported deviations of approximately 15%, whereas FFF-fabricated composites may exhibit reductions in mechanical properties of 20–35% or more due to raster orientation and processing-related factors.

Therefore, in the present study, the predicted elastic properties were adjusted based on experimental mechanical testing results. The corrected values were subsequently used in finite element simulations.

The application of an experimentally determined correction factor for CFR-PEEK containing 10 wt.% carbon fibers to predict the properties of the composite containing 30 wt.% carbon fibers is justified by the similarity of the primary mechanisms governing the formation of processing-induced defects during FDM printing.

In both cases, the same PEEK polymer matrix is used, whose thermomechanical behavior governs interlayer diffusion, sintering, and the formation of interfilament bonds [52]. It is assumed that the same printing geometry parameters, including layer height, extrusion width, and deposition path, are maintained, along with the preferential alignment of carbon fibers along the extrusion direction induced by shear flow of the polymer melt [53].

Despite the potential effects of increasing fiber content on melt viscosity and porosity, the transfer of the correction factor is considered a first-order approximation for accounting for processing-induced imperfections when predicting the elastic properties of CFR-PEEK containing 30 wt.% carbon fibers.

To account for the additional effects of the FFF printing process, the corrected predicted properties of the composite were compared with experimentally determined Young’s modulus values. The values obtained after the preliminary adjustment of the Digimat predictions were used as the computational reference.

The correction factor was calculated as the ratio of the experimentally measured Young’s modulus to the corresponding predicted value:

(15) $$K_{E}=\frac{E_{test}}{K_{calc}}$$

To develop the computational model of CFR-PEEK containing 30 wt.% carbon fibers, the initial elastic properties predicted using Digimat were adjusted using correction factors derived from the experimental mechanical tests conducted in this study. This approach accounts for the reduction in mechanical properties associated with the processing characteristics of additive manufacturing and provides elastic parameters that more closely represent the actual properties of the 3D-printed material. The calculated results and adopted correction factors are presented in Table 10.

The mechanical properties determined for the PEEK-CF30 composite containing 30 wt.% carbon fibers are consistent with findings reported in previous studies [54], investigating the mechanical behavior of carbon fiber-reinforced thermoplastic composites fabricated using FDM technology.

### 3.4. Results of Finite Element Analysis of the Bone–Plate System with Ti-6Al-4V and CFR-PEEK Implants

**Figure 16:**
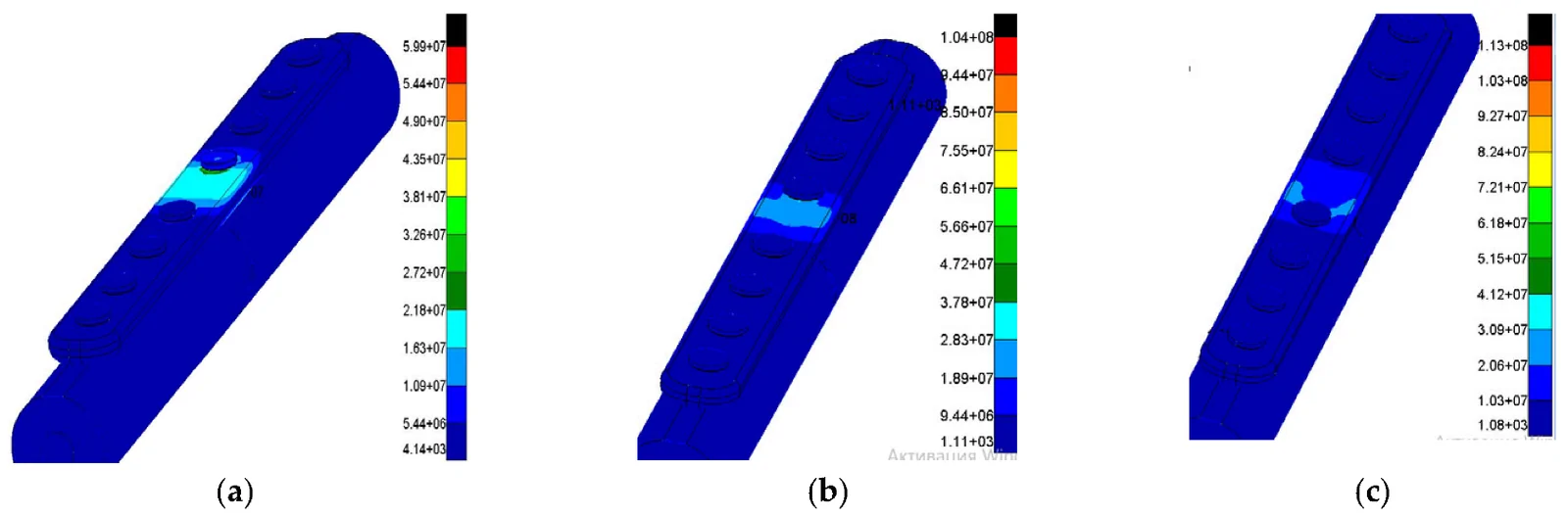
Von Mises equivalent stress distribution in the bone–plate assembly under axial compressive loading: (**a**) Ti-6Al-4V; (**b**) CFR-PEEK 10%; (**c**) CFR-PEEK 30%.

Figure 16 presents the results of finite element simulations, including the distribution of equivalent stresses calculated according to the von Mises yield criterion. The analysis considers three osteosynthesis plate configurations: a titanium alloy (Ti-6Al-4V) plate and two CFR-PEEK composite plates containing 10 and 30 wt.% carbon fibers, respectively.

The resulting stress (Figure 16) contour plots illustrate the load transfer mechanisms at the interfaces between the fixation components and bone tissue, enabling the identification of regions with the highest stress gradients and localized stress concentrations.

The obtained von Mises equivalent stress contour plots reveal pronounced stress localization near one of the central fixation screws. For the Ti-6Al-4V titanium alloy plate, the maximum equivalent stress reaches 59.9 MPa, with a relatively smooth stress distribution in the surrounding region. In the CFR-PEEK plate containing 10 wt.% carbon fibers, the maximum stress increases to 104 MPa, accompanied by a pronounced localized stress concentration. Increasing the carbon fiber content to 30 wt.% results in a further increase in the maximum stress to 113 MPa, indicating changes in the stress–strain response of the fixation construct. The increase in localized stresses associated with changes in material stiffness can be attributed to load redistribution within the bone–plate–screw assembly, including the effects of contact interactions and geometric stress concentrators around the fixation holes. It should be emphasized that von Mises equivalent stress alone is insufficient for assessing the structural integrity of orthotropic carbon fiber-reinforced polymer composites, as it does not account for direction-dependent material strength. Therefore, the subsequent analysis considers the normal and shear components of the stress tensor, followed by an assessment of composite plate strength using an anisotropic failure criterion.

**Figure 17:**
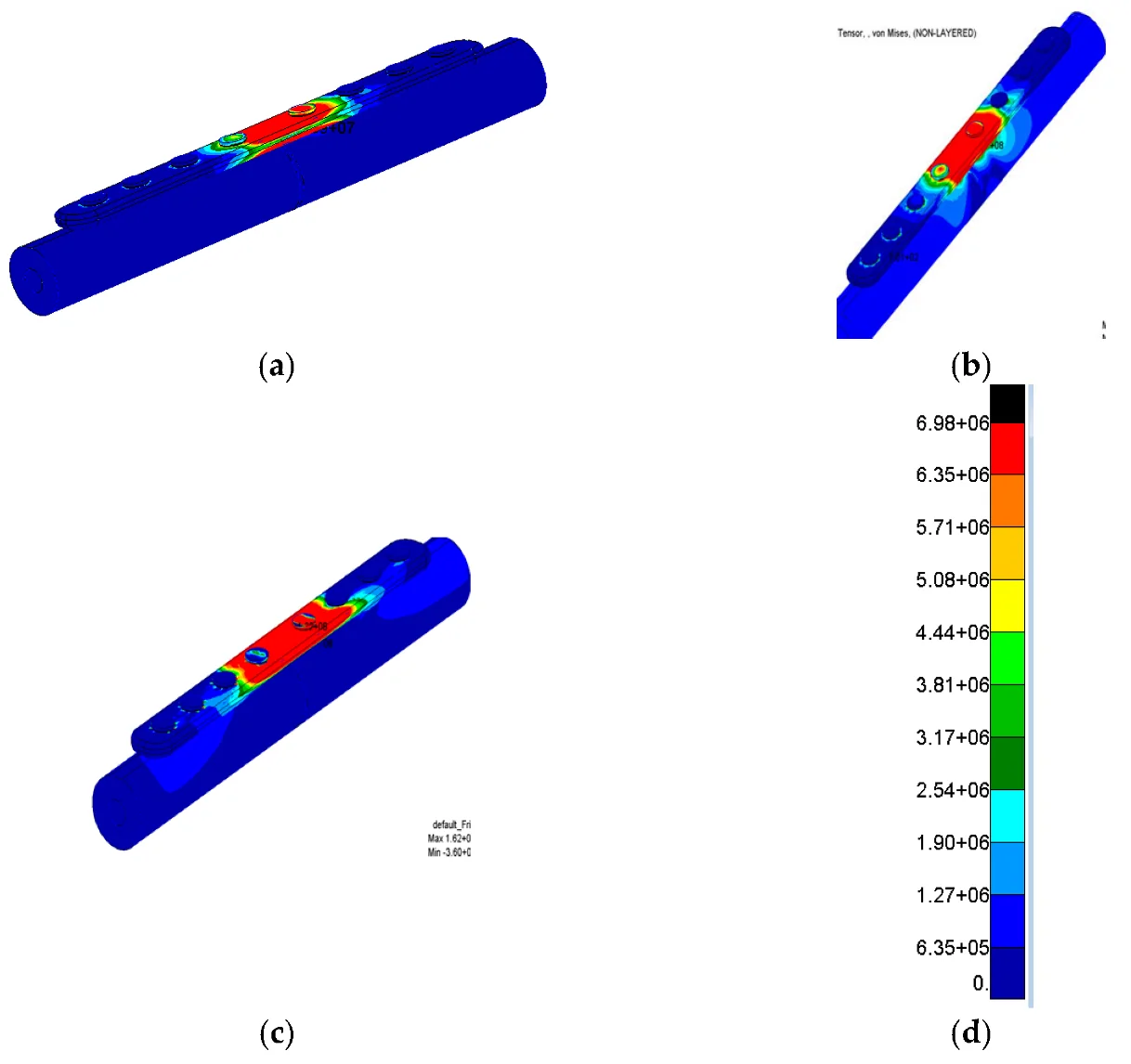
Comparative von Mises equivalent stress distributions in the bone–plate assemblies with (**a**) Ti-6Al-4V, (**b**) CFR-PEEK 10%, and (**c**) CFR-PEEK 30% fixation plates; (**d**) unified stress color scale.

For a qualitative comparison of stress distributions in osteosynthesis constructs made of Ti-6Al-4V, CFR-PEEK 10%, and CFR-PEEK 30%, all stress contour plots were presented using a unified color scale. This approach enables clear identification of differences in load transfer and stress redistribution among the investigated configurations. By eliminating the effects of automatic scaling in the visualization of simulation results (Figure 17), it allows for a direct comparison of the spatial stress distributions within the examined constructs.

A qualitative assessment of the presented stress contour plots reveals differences in load redistribution between the bone and the implant. The metallic fixation plate (Ti-6Al-4V), owing to its high stiffness, carries a substantial proportion of the applied load, whereas the use of composite materials alters the biomechanical response of the bone–plate assembly.

For the CFR-PEEK composite containing 10 wt.% carbon fibers, the predominantly light blue and turquoise regions across the plate surface indicate a more extensive distribution of stresses within the bone. This suggests a redistribution of mechanical loading along the plate. With an increase in carbon fiber content to 30 wt.%, the stress distribution in the bone becomes more localized, accompanied by a reduction in the area of highly stressed regions.

These differences suggest that the stiffness of the composite fixation plates influences load sharing between the implant and bone tissue. Such behavior may contribute to reducing the stress-shielding effect by allowing a greater proportion of the applied mechanical load to be transferred to the surrounding bone. However, confirmation of reduced stress shielding requires quantitative assessment of the stresses and strains within the bone tissue.

To quantitatively evaluate the influence of fixation plate material on load redistribution within the bone–plate assembly, a comparative analysis of von Mises equivalent stresses was performed for Ti-6Al-4V, CFR-PEEK 10%, and CFR-PEEK 30% configurations.

Unlike stress contour plots, which provide an overall representation of the stress–strain state, stress distributions extracted at selected nodes of the finite element model enable a more detailed assessment of local stress concentrations and their variation with implant mechanical properties.

The analysis was performed separately for the osteosynthesis plate and bone tissue under identical loading conditions. Particular attention was given to the relative stress levels in the implant and surrounding bone, as their distribution provides insight into the effects of fixation stiffness on load transfer and stress shielding.

**Figure 18:**
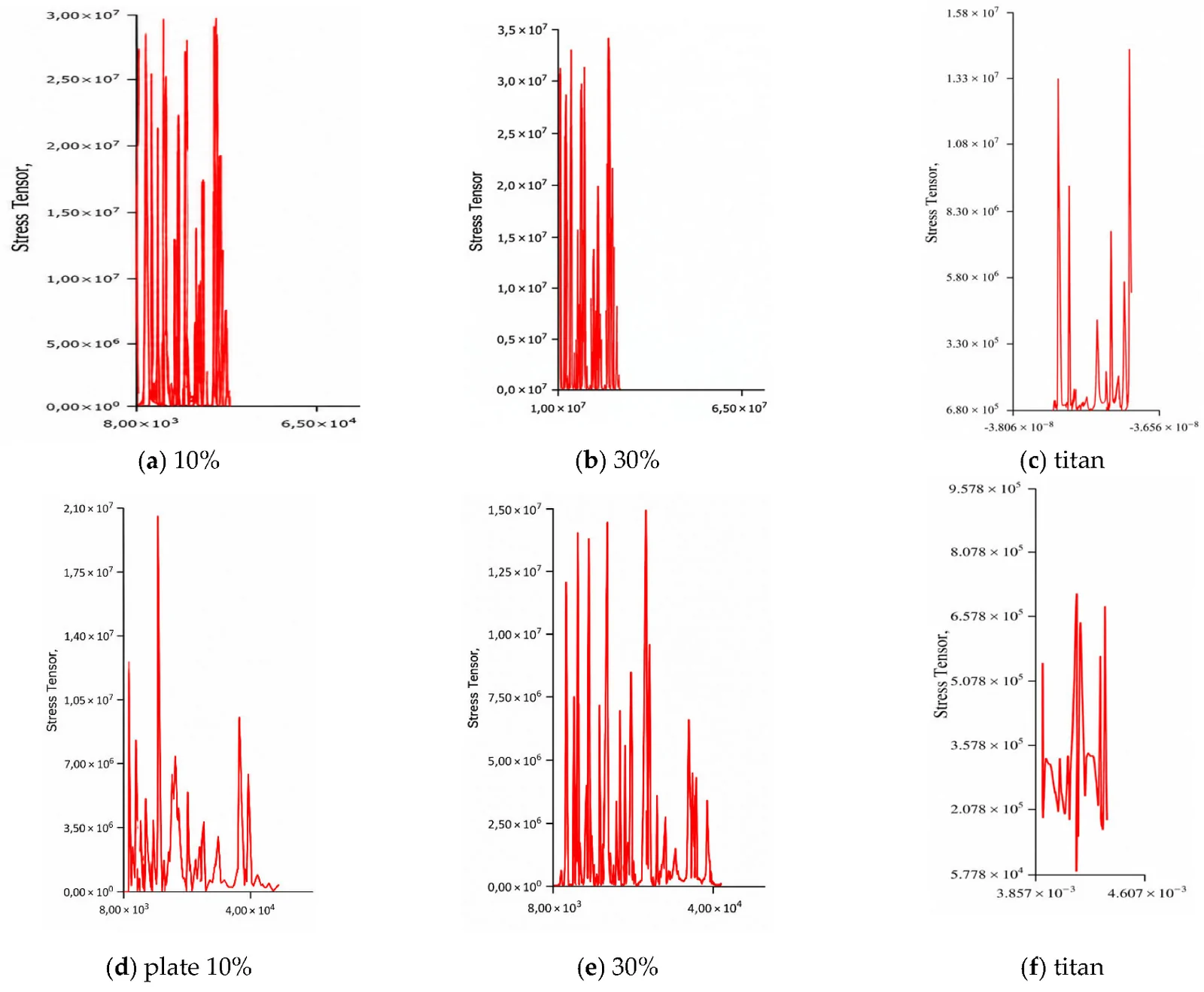
Comparative von Mises equivalent stress distributions at selected finite element nodes for CFR-PEEK 10%, CFR-PEEK 30%, and Ti-6Al-4V: (**a**–**c**) osteosynthesis plates and (**d**–**f**) corresponding bone tissue.

Comparison of the magnitude, number, and location of stress peaks allows for the identification of material-dependent mechanical responses, including stress concentration regions within the fixation plate and changes in bone loading. The results of the comparative analysis are presented in Figure 18.

Analysis of the von Mises equivalent stress distributions revealed differences in load transfer between the osteosynthesis plates and bone tissue. For all investigated materials, the maximum stresses in the fixation plates were comparable, ranging from 30 to 35 MPa. Pronounced localized stress peaks indicate the presence of stress concentration regions, primarily near the screw connections (Table 11). The most significant differences were observed in the bone tissue, where the maximum equivalent stresses reached approximately 0.8 MPa for Ti-6Al-4V, 20–21 MPa for CFR-PEEK 10%, and 16 MPa for CFR-PEEK 30%.

(16) $${SS}_{can}=\left(1-\frac{\sigma_{bone}}{\sigma_{plate}}\right)\cdot 100\%$$

where

- $\sigma_{bone}$—von Mises equivalent stress in the bone tissue, MPa;
- $\sigma_{plate}$—von Mises equivalent stress in the osteosynthesis plate, MPa.

To assess the structural integrity of osteosynthesis plates fabricated from CFR-PEEK composites containing 10 and 30 wt.% carbon fibers, the distributions of normal and shear stresses along the principal directions of the orthotropic material were analyzed. Unlike von Mises equivalent stress, the analysis of individual stress tensor components accounts for the anisotropic mechanical behavior of the composites and enables identification of the most critically loaded directions.

The finite element simulations provided the normal stress components ($\sigma_{x}$, $\sigma_{y}$, $\sigma_{z}$) and shear stress components ($\tau_{xy}$, $\tau_{yz}$, $\tau_{zx}$). Particular attention was paid to the localization of maximum stresses around the screw holes, which act as geometric stress concentrators.

The obtained results were subsequently used to evaluate the strength of the composite fixation plates using the three-dimensional generalized Tsai–Hill failure criterion, which accounts for differences in material strength along the reinforcement directions and under shear loading.

**Figure 19:**
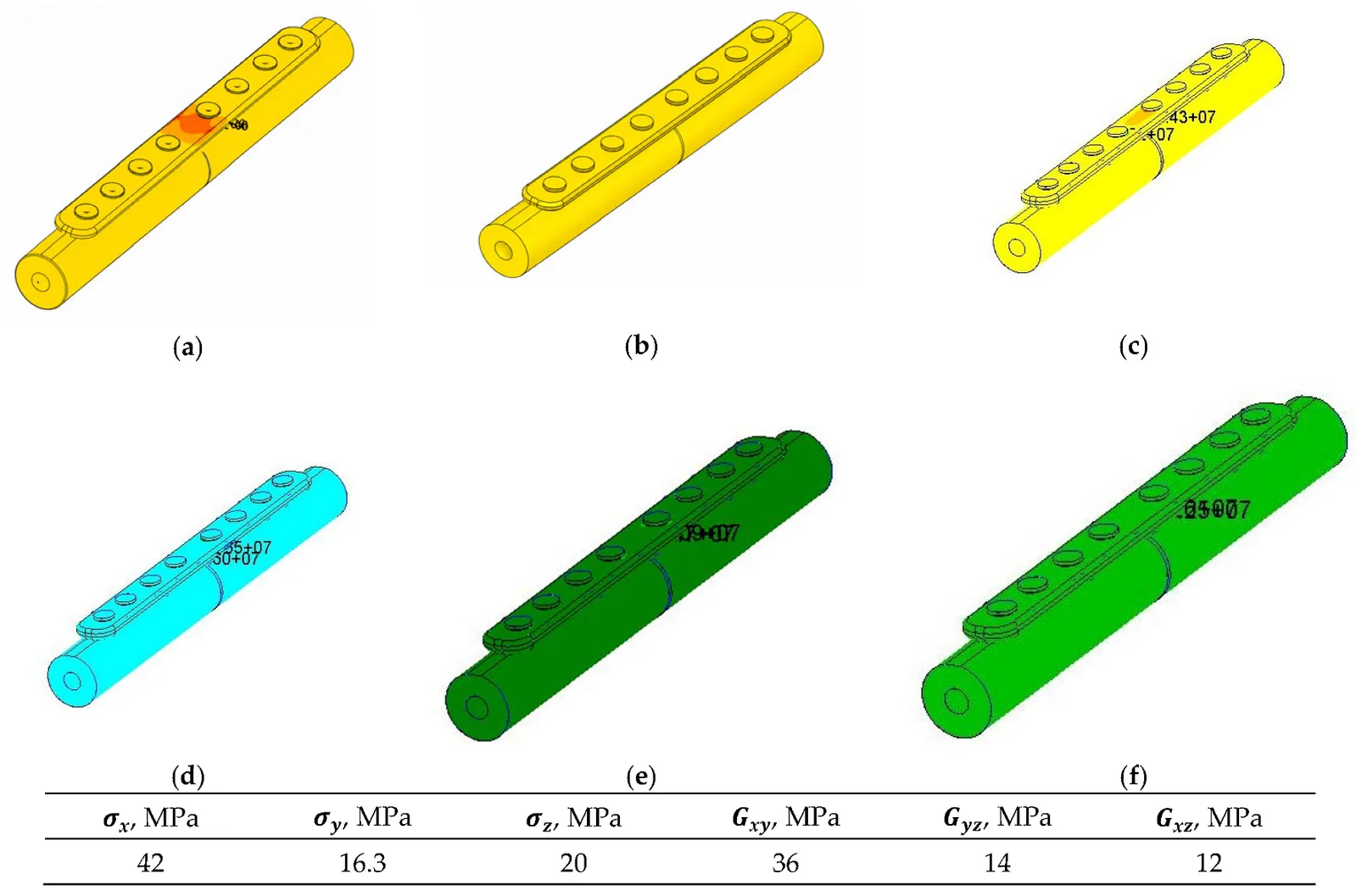
Distribution of stress components in the CFR-PEEK osteosynthesis plate containing 10 wt.% carbon fibers obtained from finite element analysis: normal stress components (**a**) σ_x_ (max 42.0 MPa), (**b**) σ_y_ (max 16.3 MPa), and (**c**) σ_z_ (max 20.0 MPa); shear stress components (**d**) τ_xy_ (max 36.0 MPa), (**e**) τ_yz_ (max 14.00 MPa), and (**f**) τ_zx_ (max 12.0 MPa).

**Figure 20:**
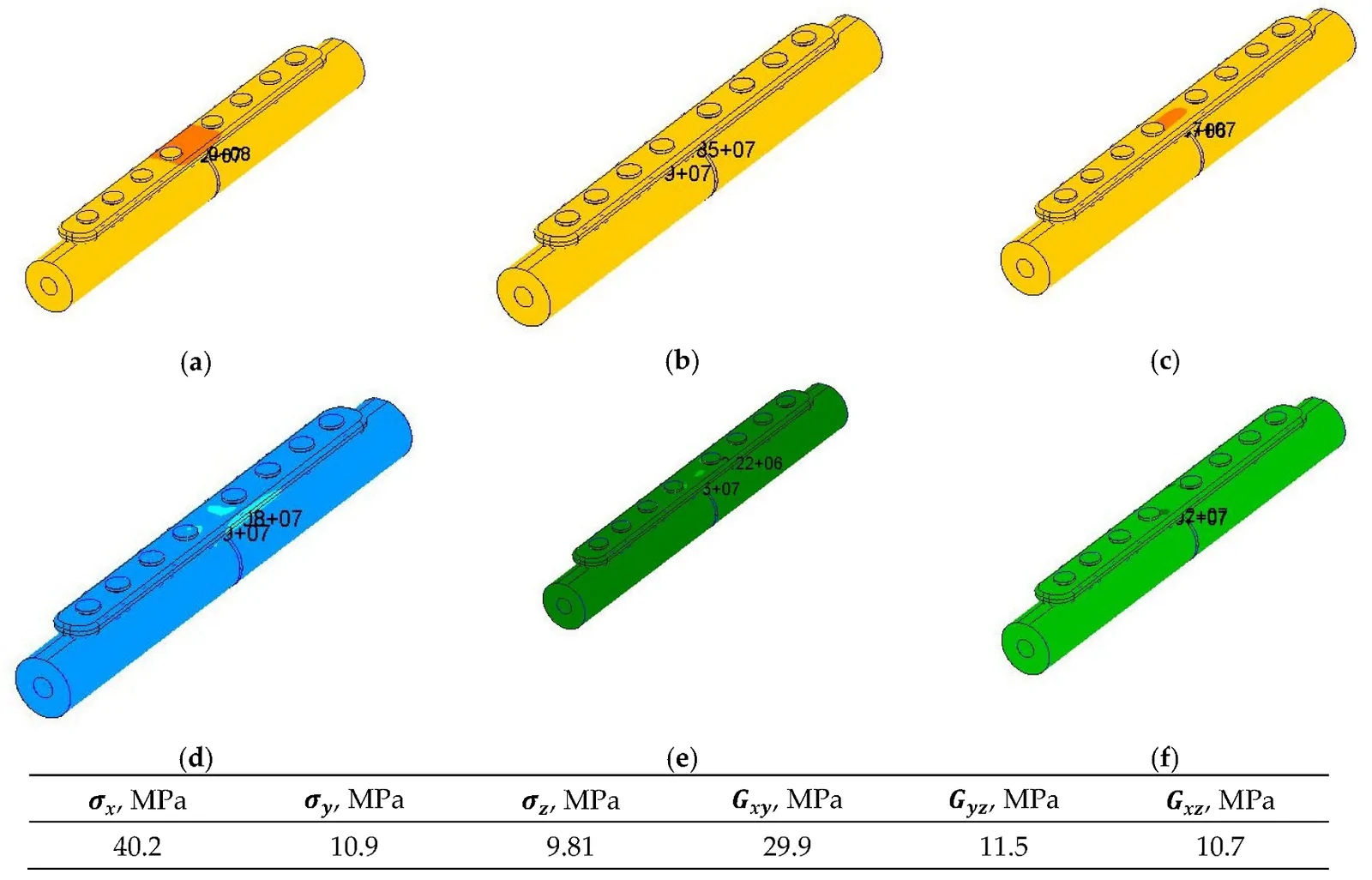
Distribution of stress components in the CFR-PEEK osteosynthesis plate containing 30 wt.% carbon fibers obtained from finite element analysis: normal stress components (**a**) σ_x_ (max 40.2 MPa), (**b**) σ_y_ (max 10.9 MPa), and (**c**) σ_z_ (max 9.81 MPa); shear stress components (**d**) τ_xy_ (max 29.9 MPa), (**e**) τ_yz_ (max 11.5 MPa), and (**f**) τ_zx_ (max 10.7 MPa).

Increasing the carbon fiber content from 10 to 30 wt.% resulted in a 2 MPa reduction in the maximum tensile stress, from 42 to 40.2 MPa, at the critical location. The observed change in the background stress distribution, from the green region (−18 MPa) to the yellow region (+19.9 MPa), suggests a redistribution of longitudinal loading across the surrounding material regions in the 30 wt.% composite. Particular attention should be paid to the red regions in the stress contour plots, which indicate critical stress concentrations localized around the edges of the central screw holes (Figure 19 and Figure 20).

The material strength values in the three orthogonal directions were determined based on experimental data, whereas the shear strength values were provisionally assumed to be 30 MPa for the preliminary assessment. The combined effects of all six stress components were taken into account in the calculations, enabling an evaluation of how closely the composite material approached its failure limit.

The resulting failure index was used to assess the structural integrity of the fixation plate and identify potentially critical regions around the screw holes.

To evaluate the strength of orthotropic CFR-PEEK composites containing 10 and 30 wt.% carbon fibers, the ultimate strength properties were determined along three mutually perpendicular directions. The strength values under normal loading for CFR-PEEK 10% were determined experimentally, whereas those for CFR-PEEK 30% were obtained from the scientific literature. The experimental determination of interlaminar and intralaminar shear strength presents significant methodological challenges associated with structural anisotropy, the characteristics of interlayer bonding, and the need for specialized testing procedures. Due to the lack of sufficient experimental data for the materials under investigation, the shear strength values were estimated using appropriate relationships derived from the principles of mechanics of materials. The resulting mechanical properties were used as input parameters for the preliminary evaluation of the failure index according to the three-dimensional Tsai–Hill failure criterion (Table 12).

For the composite containing 10 wt.% carbon fibers, the following values were adopted:

(17) $${FI}_{TH}=15.577$$

For the composite containing 30 wt.% carbon fibers, the following values were obtained:

Total Failure Index

The total failure index was calculated as the sum of the contributions from normal and shear stresses, as expressed by the following equation:

(18) $${FI}_{TH}={FI}_{normal}+{FI}_{shear}$$

The resulting total failure index is:

(19) $${FI}_{TH}=1.112$$

The shear stress components made the dominant contribution to the failure index, accounting for 0.9, whereas the contribution of the normal stress components was 0.021. This result indicates that the material may reach its failure limit under the considered loading conditions, highlighting the need to improve shear resistance in regions of localized stress concentration.

## 4. Discussion

Micromechanical modeling using Digimat-MF demonstrated that increasing the carbon fiber content from 10 to 30 wt.% increased the longitudinal Young’s modulus of CFR-PEEK from 5.30 to 25.99 GPa (a 4.90-fold increase) theoretical value, while the transverse moduli E_2_ and E_3_ increased from 3.50 to 7.79 GPa (a 2.23-fold increase). The shear moduli G_12_ and G_13_ increased from 1.49 to 3.52 GPa, whereas G_23_ increased from 1.26 to 2.63 GPa. The increase in the E_1_/E_2_ ratio from 1.51 to 3.34 confirms the enhanced orthotropic behavior of the material with increasing reinforcement content.

Experimental tensile testing of CFR-PEEK 10% revealed Young’s modulus values ranging from 1.76 to 3.10 GPa and tensile strength values of 47–54 MPa. Pareto analysis identified the interaction between extrusion temperature and layer thickness (35%), extrusion temperature (30%), and layer thickness (16%) as the dominant factors, with a combined contribution of 81%.

The reduction in tensile strength to approximately 33 MPa in the transverse (Y) and interlayer (Z) directions indicates pronounced process-induced anisotropy and highlights the significant influence of inter-raster and interlayer bonding on the mechanical strength of FDM-fabricated components [56].

The finite element analysis revealed a significant influence of CFR-PEEK mechanical properties on stress localization within the bone–plate assembly. The maximum von Mises equivalent stresses near the central fixation screw reached 59 MPa for Ti-6Al-4V, 104 MPa for CFR-PEEK 10%, and 113 MPa for CFR-PEEK 30%. Thus, replacing the titanium plate with CFR-PEEK 10% resulted in a 68.9% increase in the local maximum stress, whereas the CFR-PEEK 30% configuration exhibited an 82.6% increase. Increasing the carbon fiber content from 10 to 30 wt.% further increased the local maximum stress by 8.1%.

However, the analysis of stress distributions at selected plate nodes revealed comparable values ranging from 15 to 35 MPa for all three materials. The difference between these values and the global maximum stresses of 59–113 MPa indicates pronounced stress localization within limited regions around the fixation connections.

**Figure 21:**
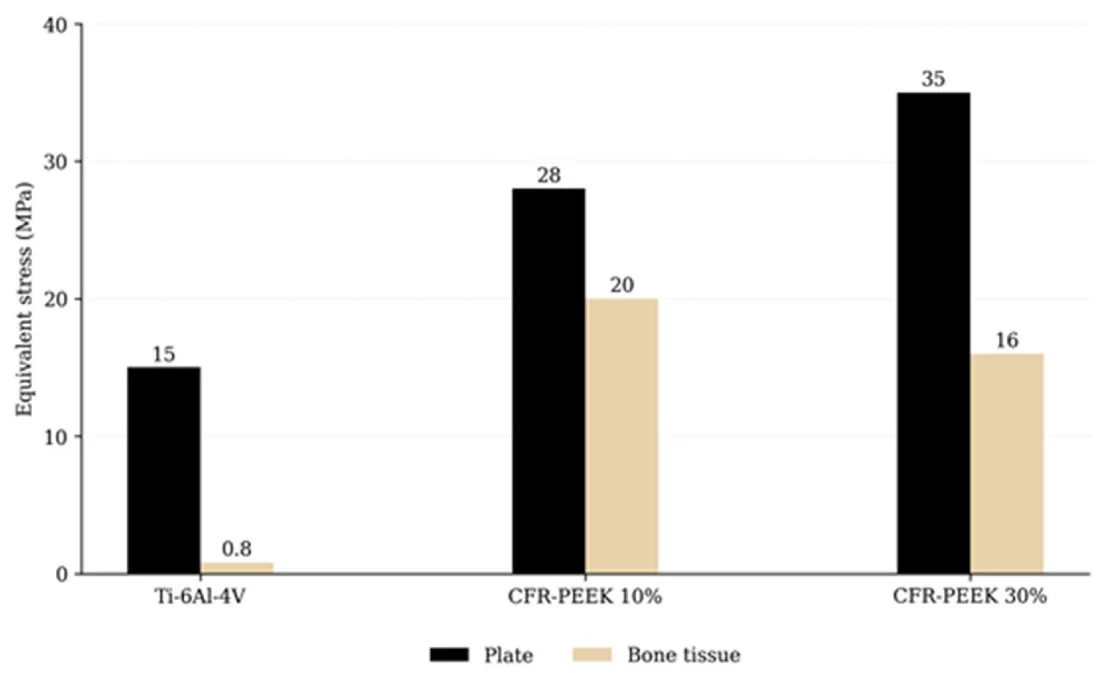
Stress distribution in the bone–plate system.

These findings demonstrate that changes in plate material properties affect not only the overall mechanical response of the fixation construct but also the intensity of localized stress concentrations. The stress distribution within the bone–plate assembly is presented in Figure 21.

The most pronounced differences were observed in the bone tissue. For the Ti-6Al-4V configuration, the maximum stresses at the selected nodes were 0.8 MPa, whereas they reached 20 MPa for CFR-PEEK 10% and 16 MPa for CFR-PEEK 30%. Thus, the use of CFR-PEEK 10% resulted in a 25-fold increase in the calculated stress value compared with the titanium fixation construct. For CFR-PEEK 30%, a 20-fold increase was observed. Increasing the carbon fiber content from 10 to 30 wt.% reduced the stresses in the bone tissue by 20%, consistent with the higher stiffness of the reinforced composite.

**Figure 22:**
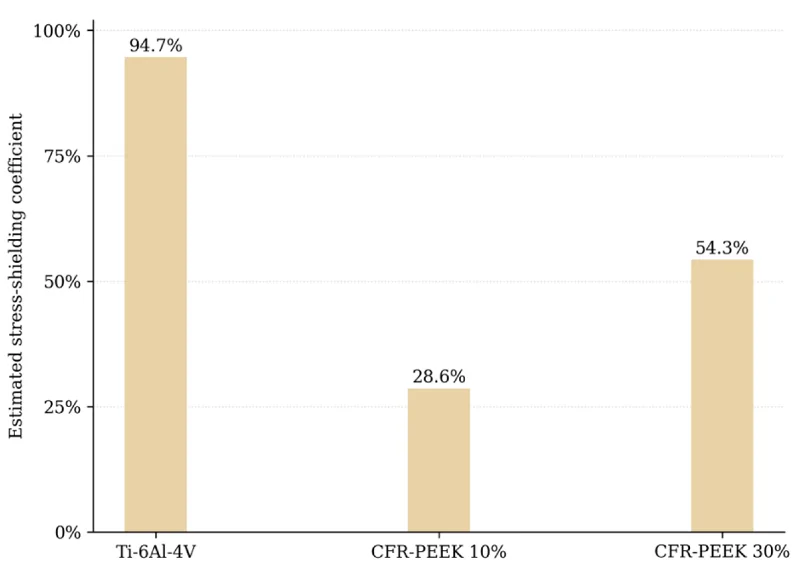
Distribution of the Estimated Stress-Shielding Index.

A similar trend (Figure 22) was observed in the assessment of the estimated stress-shielding coefficient. The corresponding values were 94.7% for Ti-6Al-4V, 28.6% for CFR-PEEK 10%, and 54.3% for CFR-PEEK 30%.

Both composite materials exhibited lower estimated stress-shielding indices compared with titanium. CFR-PEEK 10% demonstrated the lowest value, whereas increasing the carbon fiber content to 30 wt.% resulted in an increase of 18.1 percentage points. Comparison of these results with the stresses in bone tissue revealed a consistent trend: a reduction in the estimated stress-shielding index was accompanied by increased mechanical loading of the bone.

Analysis of the directional stress components showed that increasing the carbon fiber content from 10 to 30 wt.% resulted in varying degrees of reduction in the maximum normal stresses. Specifically, σ_x_ decreased from 42.0 to 40.2 MPa (by 4.3%), σ_y_ decreased from 16.3 to 10.9 MPa (by 33.1%), and σ_z_ decreased from 20.0 to 9.81 MPa (by 51.0%). The most pronounced reduction was observed along the Z-axis, indicating a significant influence of the reinforcement content on the redistribution of normal stresses within the composite osteosynthesis plate.

Meanwhile, the shear stress components also decreased with increasing carbon fiber content from 10 to 30 wt.%. Specifically, τ_xy_ decreased from 36.0 to 29.9 MPa (by 16.9%), τ_yz_ from 14.0 to 11.5 MPa (by 17.9%), and τ_zx_ from 12.0 to 10.7 MPa (by 10.8%). The most pronounced reduction was observed in the τ_yz_ component. Thus, increasing the reinforcement content resulted in a simultaneous reduction in both normal and shear stress components, indicating a redistribution of stresses within the composite osteosynthesis plate.

A three-dimensional Tsai–Hill failure criterion was employed for the preliminary assessment of the structural integrity of the composite fixation plates. Based on the assumed directional strength limits and shear strength values, the calculated failure index (FI) was 15.507 for CFR-PEEK 10% and 1.112 for CFR-PEEK 30%, corresponding to a reduction of approximately 97.3%. This substantial reduction indicates an improvement in the predicted failure resistance of the composite with a higher degree of reinforcement under the assumed loading conditions. While CFR-PEEK 10% significantly exceeded the critical threshold of FI = 1, CFR-PEEK 30% remained closer to this limit, suggesting that the latter satisfies the adopted strength criterion under the specified loading conditions.

The combined analysis revealed a trade-off between stiffness, mechanical load transfer to bone tissue, and the structural strength of the composite implant. CFR-PEEK 10% exhibited a lower estimated stress-shielding coefficient of 28.6%, but a higher calculated failure index (FI = 15.507). Increasing the carbon fiber content to 30 wt.% reduced the failure index to 1.112, while simultaneously increasing the estimated stress-shielding coefficient to 46.7%. Nevertheless, both composite materials demonstrated an advantage over Ti-6Al-4V in terms of the adopted indicator of mechanical load transfer to bone tissue.

Thus, increasing the carbon fiber content enhances the predicted stiffness and improves the structural strength of CFR-PEEK under the investigated loading conditions, while simultaneously altering the load distribution between the implant and surrounding bone tissue. These findings indicate that the selection of an appropriate reinforcement content for osteosynthesis plates should be based on a comprehensive assessment of stress shielding, directional stress components, and structural strength in the regions surrounding fixation holes, rather than solely on the longitudinal Young’s modulus.

The analysis based on the Tsai–Hill failure criterion showed that the calculated failure index for CFR-PEEK 10% (FI = 15.577) significantly exceeded the critical threshold of FI = 1, whereas the CFR-PEEK 30% composite (FI = 1.112) practically satisfied the adopted strength criterion. In both materials, the maximum stresses were predominantly localized around the fixation holes, particularly at the plate–screw contact interfaces.

To reduce stress concentrations, it is advisable to optimize the geometry of the fixation holes, introduce smooth geometric transitions and chamfers, and consider the use of metallic bushings. Increasing the plate thickness is undesirable due to the anatomical constraints of the radial diaphysis [4]. Thus, CFR-PEEK 30% demonstrates more favorable predicted strength characteristics; however, further numerical and experimental investigations are required to confirm its structural reliability.

## 5. Conclusions

The obtained results highlight the importance of accounting for the orthotropic mechanical properties of carbon fiber-reinforced polymer composites in the design of osteosynthesis plates. It was established that variations in carbon fiber content significantly affect not only the longitudinal stiffness of the material but also the distribution of normal and shear stresses, load transfer to bone tissue, and the predicted structural strength of the implant. These findings demonstrate the limitations of conventional approaches based solely on isotropic material properties.

Although CFR-PEEK 30% specimens were neither fabricated nor experimentally tested in the present study, their mechanical properties were predicted using Digimat-MF by applying correction factors derived from experimental data obtained for CFR-PEEK 10%. Thus, the results for CFR-PEEK 30% are computational predictions and require further experimental validation. The proposed integrated approach, combining micromechanical modeling in Digimat-MF, experimental evaluation of the effects of FDM printing, and finite element analysis incorporating orthotropic material behavior, provides a foundation for the predictive design of composite osteosynthesis plates. This approach enables the prediction of implant mechanical behavior at the design stage, assessment of the effects of fiber content and orientation, identification of critical stress concentration regions, and informed selection of material properties.

The practical significance of this approach is particularly evident when using relatively accessible composite materials based on thermoplastic polymers reinforced with short carbon fibers, which may offer a cost-effective alternative to more expensive high-performance reinforced materials. Furthermore, the possibility of manufacturing such structures using FDM printing expands the potential for patient-specific implant fabrication with reduced material consumption and tailored mechanical properties.

Thus, the scientific contribution of this study lies in the development and application of an integrated experimental–computational approach for predicting the mechanical behavior of orthotropic composite osteosynthesis plates. The obtained results provide a basis for further structural optimization, considering mechanical strength requirements, stress-shielding reduction, and anatomical constraints. Further experimental validation and assessment of long-term structural reliability are required before the proposed approach can be considered for clinical applications.

## Figures and Tables

**Table 1:** Initial Material Properties of PEEK-CF Composite Constituents.

Parameter	PEEK Matrix	Carbon Fiber (CF)
Young’s modulus, E (GPa)	3.70	230
Poisson’s ratio, ν	0.38	0.20
Density, ρ (g/cm^3^)	1.30	1.80
Material model	Isotropic	Isotropic linear elastic
Role in the composite	Matrix phase	Reinforcing phase

**Table 2:** Parameters of the PEEK-CF 10% and PEEK-CF 30% Composite Modeling Configurations.

Parameter	PEEK-CF10	PEEK-CF30
Carbon fiber weight fraction	10%	30%
PEEK weight fraction	90%	70%
Matrix material	PEEK	PEEK
Reinforcing phase	Short carbon fibers	Short carbon fibers
$E_{PEEK}$	3.70 GPa	3.70 GPa
$E_{CF}$	230 GPa	230 GPa
Aspect ratio	20	20
Fiber aspect ratio (L/D)	$\left[\begin{array}{ccc} 0.8 & 0 & 0\\ 0 & 0.1 & 0\\ 0 & 0 & 0.1 \end{array}\right]$	$\begin{array}{ccc} 0.8 & 0 & 0\\ 0 & 0.1 & 0\\ 0 & 0 & 0.1 \end{array}$

**Table 3:** Parameters of the PEEK-CF 10% Composite Modeling Configurations.

No.	Nozzle Temperature (°C)	Layer Thickness (mm)	Printing Speed (mm/s)
1	380	0.16	20
2	380	0.16	40
3	400	0.16	20
4	400	0.16	40
5	380	0.28	20
6	380	0.28	40
7	400	0.28	20
8	400	0.28	40

**Table 4:** Mechanical Properties of the Materials Used in Finite Element Analysis.

Material	Young’s Modulus (GPa)	Poisson’s Ratio
Cortical bone	24	0.30
Ti-6Al-4V	110	0.34
CFR-PEEK 10%	Orthotropic *	Orthotropic *
CFR-PEEK 30%	Orthotropic *	Orthotropic *

* Note: The effective orthotropic elastic properties of CFR-PEEK containing 30% carbon fibers were determined using micromechanical modeling in DIGIMAT, followed by adjustments to account for the processing characteristics of FFF printing. The methodology and results are presented in Section 2.2.

**Table 5:** Input Parameters of the PEEK Matrix and Carbon Fibers for CFR-PEEK Modeling in Digimat-MF.

Parameter	CFR-PEEK 10%	CFR-PEEK 30%
Matrix material	PEEK	PEEK
Reinforcement phase	Short CF	Short CF
CF mass fraction, %	10	30
PEEK mass fraction, %	90	70
Young’s modulus of PEEK, GPa	3.70	3.70
Poisson’s ratio of PEEK	0.38	0.38
Young’s modulus of CF, GPa	230	230
Poisson’s ratio of CF	0.20	0.2
Fiber diameter, mm	0.00866	0.00866
Aspect ratio	20	20
Calculated fiber length, mm	0.1732	0.1732
Fiber orientation tensor	diag(0.8; 0.1; 0.1)	diag(0.8; 0.1; 0.1)
Homogenization model	Mori–Tanaka	Mori–Tanaka

**Table 6:** Theoretical Mechanical Properties of the CFR-PEEK Composite Containing 10 wt.% Carbon Fibers.

Property	Value (GPa)
$\mathrm{Young}’s\;\mathrm{Modulus}\;E_{1}.$	5.3
$\mathrm{Young}’s\;\mathrm{Modulus}\;E_{2}.$	3.5
$\mathrm{Young}’s\;\mathrm{Modulus}\;E_{3}.$	3.5
$\mathrm{Young}’s\;\mathrm{Modulus}\;G_{12}.$	1.49
$\mathrm{Young}’s\;\mathrm{Modulus}\;G_{23}$	1.26
$\mathrm{Young}’s\;\mathrm{Modulus}\;G_{13}$	1.49
Poisson’s Ratio $\nu_{12}$	0.38
Poisson’s Ratio $\nu_{23}$	0.41
Poisson’s Ratio $\nu_{13}$	0.38

**Table 7:** Theoretical Mechanical Properties of the CFR-PEEK Composite Containing 30 wt.% Carbon Fibers.

Property	Value (GPa)
$\mathrm{Young}’s\;\mathrm{Modulus}\;E_{1}.$	25.99
$\mathrm{Young}’s\;\mathrm{Modulus}\;E_{2}.$	7.79
$\mathrm{Young}’s\;\mathrm{Modulus}\;E_{3}.$	7.79
$\mathrm{Young}’s\;\mathrm{Modulus}\;G_{12}.$	3.52
$\mathrm{Young}’s\;\mathrm{Modulus}\;G_{23}$	2.63
$\mathrm{Young}’s\;\mathrm{Modulus}\;G_{13}$	3.52
Poisson’s ratio $\nu_{12}$	0.38
Poisson’s ratio $\nu_{23}$	0.38
Poisson’s ratio $\nu_{13}$	0.47

**Table 8:** Experimental mechanical properties of the CFR-PEEK 10% composite.

No.	Young’s Modulus (E)	Ultimate Tensile Strength (Rm)
-	[MPa]	[MPa]
Specimen 1	1910.22	50
Specimen 2	3100.14	56
Specimen 3	1800.48	48
Specimen 4	1560.58	47
Specimen 5	1850.02	49
Specimen 6	1950.76	50
Specimen 7	1820.55	48
Specimen 8	2905.80	52

**Table 9:** Comparison of Theoretical and Experimental Mechanical Properties of CFR-PEEK 10 wt.%.

Mechanical Property	DIGIMAT, GPa	Experimental, GPa	Correction Factor, $k_{i}$
$E_{x}$	5.30	3.10	0.585
$E_{y}$	3.50	2.10	0.600
$E_{z}$	3.50	2.10	0.600
$G_{xy}$	1.49	0.89	0.597
$G_{zz}$	1.49	0.75	0.503
$G_{yz}$	1.26	0.89	0.706

**Table 10:** Theoretical and technologically adjusted orthotropic elastic properties of 30 wt.% CFR-PEEK composite.

Mechanical Property	DIGIMAT	Corrected Values (k = 0.60)
$E_{1},\;GPa$	25.99	15.594
$E_{2},\;GPa$	7.79	4.674
$E_{3},\;GPa$	7.79	4.674
$G_{12}\;GPa$	3.52	2.112
$G_{23\;}GPa$	2.63	1.578
$G_{13}\;GPa$	3.52	2.112
$\nu_{12}$	0.38	0.38
$\nu_{23}$	0.38	0.38
$\nu_{13}$	0.47	0.47

**Table 11:** Comparison of Equivalent Stresses and Relative Stress-Shielding Indices for Ti-6Al-4V and CFR-PEEK Osteosynthesis Plates.

Material	Plate Stress, MPa	Bone Stress, MPa	Relative Stress-Shielding Index, %
Ti-6Al-4V	15	0.8	94
CFR-PEEK 10%	28	20	28.57
CFR-PEEK 30%	35	16	54.29

**Table 12:** Strength parameters of CFR-PEEK composites with 10 and 30 wt.% carbon fiber content used for the Tsai–Hill failure criterion.

Composite	Stress	Author
CFR-PEEK 10%	$\left[\begin{array}{ccc} 56 & 12 & 5.5\\ 12 & 33 & 12\\ 5.5 & 12 & 32 \end{array}\right]$	Experimental–Computational.
CFR-PEEK 30%	$\left[\begin{array}{ccc} 170 & 34 & 45\\ 34 & 102 & 25\\ 45 & 25 & 52 \end{array}\right]$	Csenge Tóth, Ábris Dávid Virág [55]

## Data Availability

The raw data supporting the conclusions of this article will be made available by the authors on request.
